# Drivers of population dynamics and juvenile mortality in Northwest Atlantic harp seals

**DOI:** 10.1002/eap.70184

**Published:** 2026-02-20

**Authors:** M. Tim Tinker, Garry B. Stenson, Arnaud Mosnier, Joanie Van de Walle, Shelley L. C. Lang, Mike O. Hammill

**Affiliations:** ^1^ Ecology & Evolutionary Biology University of California Santa Cruz California USA; ^2^ Nhydra Ecological Consulting Head of St. Margaret's Bay Halifax Nova Scotia Canada; ^3^ Fisheries and Oceans Canada, Northwest Atlantic Fisheries Centre St. John's Newfoundland and Labrador Canada; ^4^ Fisheries and Oceans Canada Maurice Lamontagne Institute Mont Joli Québec Canada

**Keywords:** abundance, Bayesian model, climate change, harp seal, integrated population model, juvenile survival, *Pagophilous groenlandicus*, population dynamics

## Abstract

Human‐induced threats to terrestrial and marine wildlife are on the rise, and while some species face a single major threat, others face multiple concurrent threats. Harp seals, an abundant pinniped in the North Atlantic that was historically depleted by human harvest, are one such species. Although commercial and subsistence harvests remain a significant source of mortality, in recent decades their environment has undergone significant changes, which could also impact population dynamics. Inferring the relative importance of various threats as drivers of population dynamics can be challenging, particularly for marine species where monitoring abundance is difficult: the use of integrated population models (IPMs), which leverage multiple data sources to parameterize process‐based models of population dynamics, provides one solution. We developed a hierarchical Bayesian IPM with which to explore the shifting roles of anthropogenic and environmental factors in driving trends. We used a competing hazards formulation for survival, enabling the partitioning of mortality into multiple discreet causes and allowing us to assess variation in hazards over 7 decades (1952–2019). We fit the model to available data on pup production, fecundity, age structure, human removals, and environmental conditions. We conducted a Bayesian life stage simulation analysis (LSA) to compare the contributions of various hazards to variation in population growth. We found that harvests of young of the year (YOY) and adults were the primary contributors to variation in trends from 1951 to 1982; however, after 1983, the relative importance of harvest mortality decreased while the impacts of natural mortality increased, especially for YOY. Since 2000, the impacts of YOY mortality from ice cover anomalies have become one of the strongest drivers of trends, while harvest mortality has declined. Based on current climate models, which project warmer water and decreasing ice cover, we expect continued high levels of YOY mortality from environmental factors such as deteriorating ice conditions. These climate‐related hazards are likely to become the dominant drivers of population dynamics in coming decades, which will in turn affect sustainable harvest levels for both Canada and Greenland. Our model will provide a useful tool for exploring future scenarios of climate impacts and management strategies.

## INTRODUCTION

Anthropogenic disturbances are causing major threats to biodiversity, with overexploitation, climate change, habitat loss, invasive species, and pollution being the five most pervasive human‐induced threats worldwide (Living Planet Report, 2016). While some species may face a single major threat that is easily identifiable, many others face multiple concurrent threats (Pelletier & Coltman, 2018) that can act synergistically to drive population trajectories (Brook et al., 2008). Disentangling the relative contribution of multiple threats to population fluctuations over time is of great importance as it may help to explain the drivers of population trends. It may also help to identify the key drivers to monitor and where to direct conservation or management efforts in the future (Brook et al., 2008). However, this task is challenging since threats can exert varying levels of impact over time, the responses to different drivers may or may not be correlated (Isaac & Cowlishaw, 2004), and, for long‐lived species, long‐term ecological data may be lacking.

For many marine mammals, exploitation through large‐scale commercial harvesting has been a primary driver of historical population declines (Lotze & Worm, 2009). International conservation efforts since the latter half of the 20th century (e.g., International Whaling Commission, United States Marine Mammal Protection Act) (Lavigne, 2006) have contributed to the recovery of some species, but for many species, the magnitude of recovery compared to historical baselines remains low (Lotze et al., 2017; Lotze & Worm, 2009). In addition, unintended human‐caused mortality (due primarily to bycatch in fisheries) and environmental degradation (e.g., habitat loss and collapse of prey populations from overfishing) remain significant threats, aggravated by the emerging impacts of climate change (Nelms et al., 2021). For depleted marine mammal populations, as with many other taxa, recovery (or lack thereof) can be determined by a combination of factors such as distribution limits, intrinsic life history traits, and extrinsic factors such as habitat disturbance, environmental drivers, anthropogenic mortality, and management efforts (Lotze et al., 2017; Magera et al., 2013). Understanding how these different factors may impact marine mammal populations is crucial to conservation and management (McHuron et al., 2023); yet, their relative influence on population trends remains poorly known.

Integrated population models (IPMs) combine data from several sources into a single model to allow the simultaneous estimation of demographic parameters and the interpretation and projection of population trajectories (Frost et al., 2023). They are especially useful when survey data alone are insufficient to estimate precise vital rates and abundance, as their construction allows leveraging information from multiple data sources (Abadi et al., 2010). IPMs can facilitate understanding of mechanisms of population growth and decline (e.g., Mosnier et al., 2015). They can also be used to predict future population trajectories in novel circumstances, for example, by testing various scenarios of exploitation to help guide management actions (e.g., ringed seals, *Phoca hispida*, Ersalman et al., 2025), or to infer the demographic impacts of climate change (e.g., Emperor penguin, *Aptenodytes forsteri*; Jenouvrier et al., 2025) and incidence of extreme climate events (e.g., black‐browed albatrosses, *Thalassarche melanophris*; Ventura et al., 2023). A key challenge for wildlife conservation in changing environments is understanding the relative importance of different drivers of vital rates. One of the major appeals of IPMs lies in their potential to explore relationships among multiple sources of mortality such as harvest, environmental stressors, changes in food resources, predation, and longer term factors such as climate change (Riecke, Lohman, et al., 2022; Riecke, Sedinger, et al., 2022; Roberts et al., 2023). This feature of IPMs can be particularly important in the case of marine mammal populations facing multiple threats (Ersalman et al., 2025; Joly et al., 2009; Mosnier et al., 2015; Stewart et al., 2023; Tinker et al., 2021).

**FIGURE 1:**
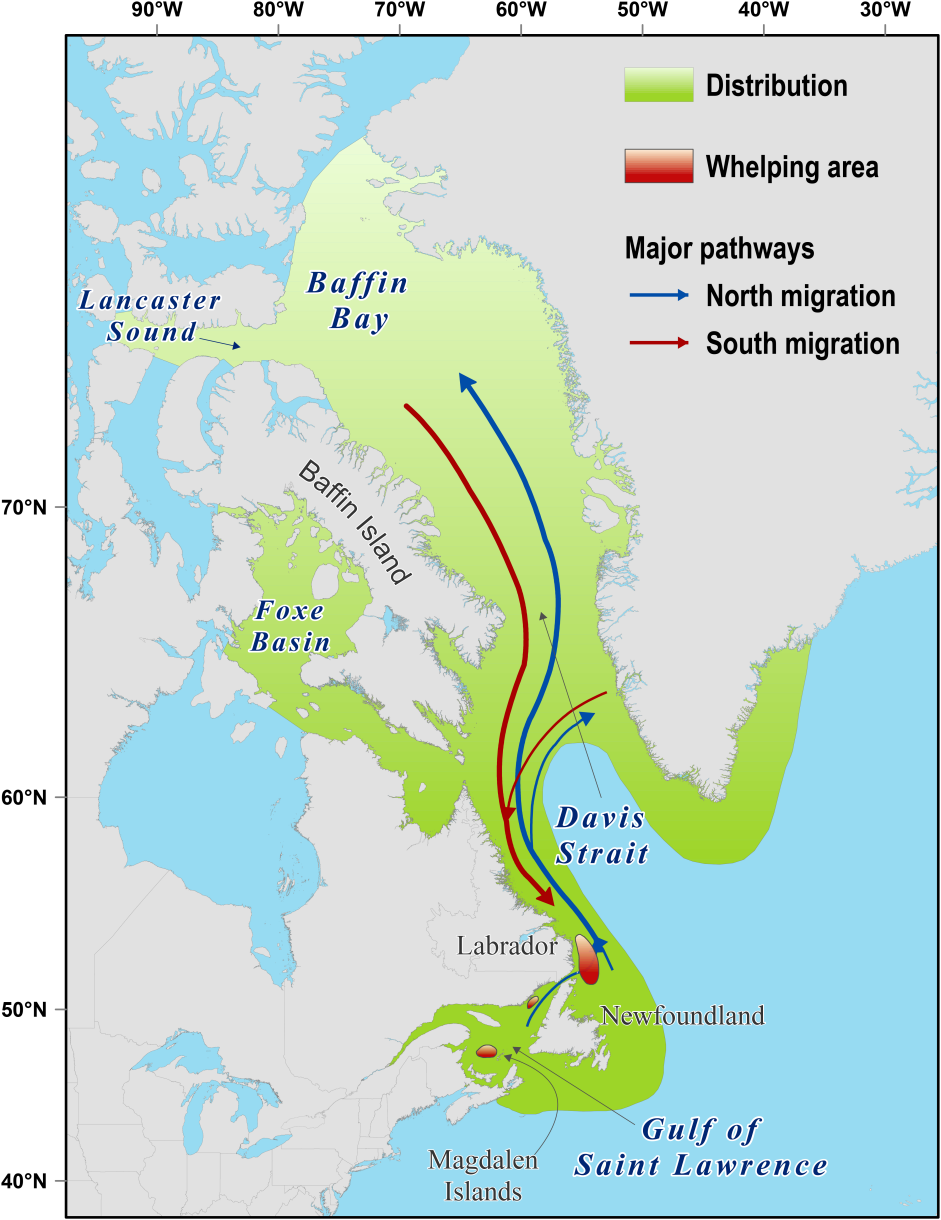
Distribution of Northwest Atlantic harp seal population along with location of the three whelping areas (red patches) and major migratory pathways.

Harp seals (*Pagophilus groenlandicus*) are an ice‐dependent, migratory pinniped found over continental shelf regions of the North Atlantic and the adjacent Arctic Ocean (Sergeant, 1991). They are the most abundant pinniped in the North Atlantic, with three populations distinguished by their whelping (pupping) location: the White Sea/Barents Sea, the Greenland Sea, and the Northwest Atlantic (NWA; Stenson, Haug, & Hammill, 2020). The NWA population, which whelps on the pack ice in Atlantic Canada (Figure 1), is the largest of the three populations (Stenson, Haug, & Hammill, 2020). This population summers in the eastern Canadian Arctic and off west Greenland, and then migrates southward to overwinter on the Newfoundland Shelf (Front) and in the Gulf of St. Lawrence (Gulf). Harp seals are an ice‐obligate species that whelps on drifting, first‐year pack ice during February–March in the Gulf and at the Front. Pups are nursed for approximately 12 days, after which the female weans her pup, mates, and disperses to feed (Sergeant, 1991). Following weaning, pups need stable ice for 4–6 weeks as they transition to nutritional independence and develop the physiological capacity for diving (Burns et al., 2007; Sergeant, 1991). If ice cover or thickness is not sufficient to withstand wave action and storms during the nursing and post‐weaning periods, the ice can break up leading to high levels of pup mortality (Stenson & Hammill, 2014). On the Newfoundland Shelf, variation in the mid‐winter ice extent and the timing of ice retreat have also been shown to influence the biomass of harp seal prey and have been linked to changes in body condition and variability in reproductive rates (Buren et al., 2014; Stenson et al., 2016; Stenson, Buren, & Sheppard, 2020).

The NWA is undergoing a period of significant change with modifications occurring in a variety of components including temperature, ocean circulation and sea ice cover (IPCC, 2021). If the current climate trends persist, the NWA harp seal population will likely face a substantial reduction in ice cover (Han et al., 2015, 2019). Understanding how removals and changes in environmental conditions affect life history parameters and ultimately population dynamics will be essential for the effective management of this population.

In this study, we develop an IPM to examine the relative impacts of harvesting and variation in environmental conditions on the dynamics of the NWA harp seal population over the last 70 years. Our primary objectives are to (1) describe trends in population abundance, (2) analyze the demographic processes responsible for driving those trends, and (3) explore the degree to which various environmental and anthropogenic factors affect demographic vital rates, and how those effects have changed over time. Our model is designed to provide data‐driven estimates of age‐specific survival and fecundity, density‐dependent effects, mortality from ice anomalies and human removals, and effects of environmental conditions on fecundity and survival. We then retrospectively investigate how the relative contribution of different sources of mortality has varied, measuring their impact on past fluctuations in population abundance via a simulation‐based sensitivity analysis. Model fitting was conducted using a hierarchical Bayesian state‐space approach that allows for more robust characterization of uncertainty, disentanglement of process error from observer error (Ahrestani et al., 2013), and incorporation of multiple data sources with different distributions and variance structures (Buckland et al., 2004; Wang, 2009; Williams et al., 2017).

## METHODS

### Data sources

**FIGURE 2:**
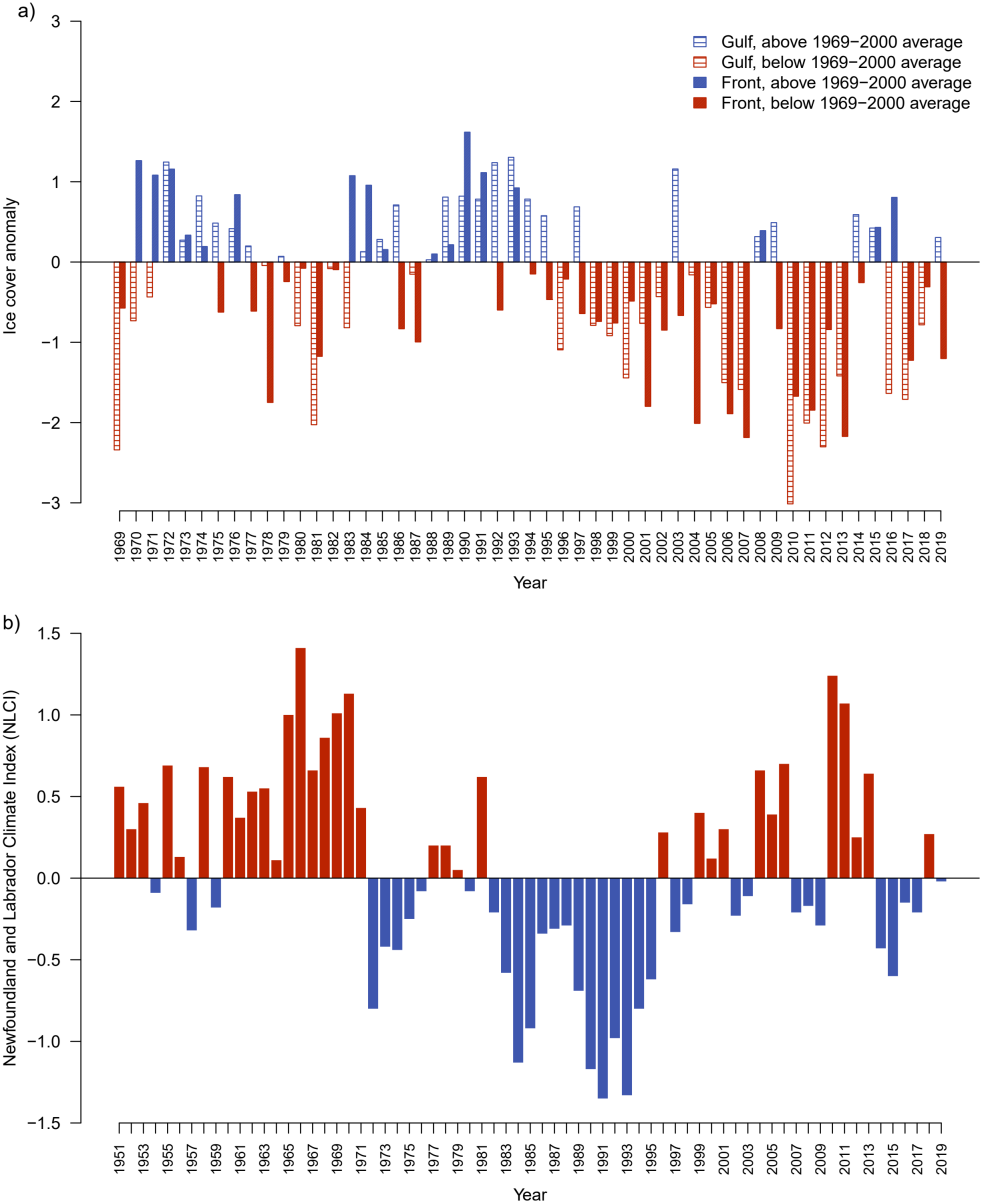
Trends in key environmental datasets over the study period. (a) Ice anomalies using mean first‐year ice cover during 1969–2000 as the baseline year. Positive anomalies mean more ice cover, and negative anomalies mean less ice cover. Data from the Canadian Ice Service of Environment and Climate Change Canada for the areas Gulf of St Lawrence and southern Labrador. (b) Newfoundland and Labrador Climate Index (NLCI) for 1951–2019 developed by Cyr and Galbraith (2021). Positive anomalies reflect warmer conditions, while negative anomalies reflect cooler conditions.

**FIGURE 3:**
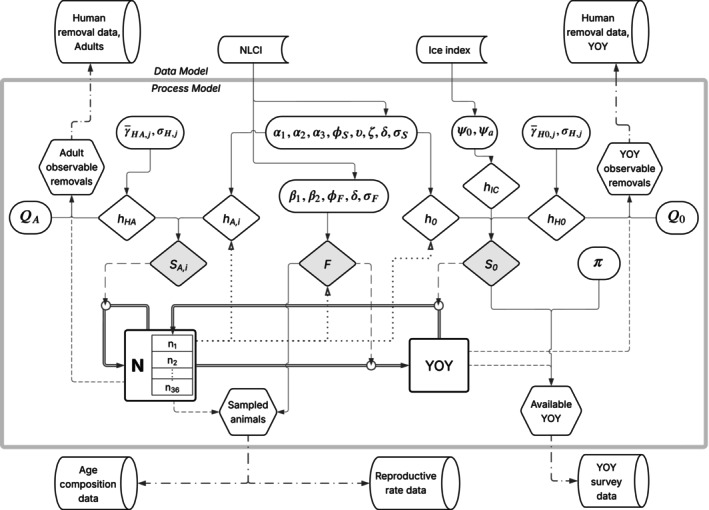
Conceptual schematic of the integrated population models, showing relationships between elements of the process model (inside gray outline) and the data model (outside gray outline). Symbols can be interpreted as follows: Rectangular shapes represent latent dynamic variables of the process model; hexagon shapes represent observable variables derived from latent variables; diamond shapes represent vital rates (shaded) or the instantaneous hazards that contribute to those vital rates (unshaded); oval shapes enclose the process model parameters that together determine the value of each hazard or vital rate; cylinder shapes represent empirical datasets used for model fitting; half cylinders represent environmental data inputs to the model; double lines represent demographic transitions between latent variables occurring at each annual timestep, with arrows pointing in the direction of the transition; solid lines represent functional relationships between environmental variables, parameters, vital rates, and observable variables, with arrows pointing in the direction of causation; long‐dashed lines indicate vital rates acting on demographic transitions; dotted lines represent density‐dependent feedbacks between abundance and vital rates or hazards; short‐dashed lines represent relationships between latent and observable variables; and dash‐dot lines represent probabilistic relationships between observable variables of the process model and empirical datasets.

The model relies on six sources of data: (1) pup production estimates from 1951 to 2017 (Appendix S1: Table S1); (2) reproductive rates derived from samples collected between October and February, 1979–2019, around Newfoundland and southern Labrador under licenses issued by the Department of Fisheries and Oceans (DFO) Canada (Appendix S1: Table S2; Stenson, Haug, & Hammill, 2020); (3) age composition data from seals collected for reproductive rates and as part of other sampling programs carried out by DFO since 1979 (Appendix S1: Table S3); (4) reported human‐caused deaths (removals) from four sources: (i) the commercial harvest in Atlantic Canada; personal and subsistence hunts in (ii) the Canadian Arctic, and (iii) Greenland; and (iv) incidental by‐catch in commercial fishing gear (Appendix S1: Table S4; Stenson, 2010; Stenson & Upward 2020); (5) an ice index (IC) calculated from government ice cover data and used to provide a measure of ice availability and stability for each year of the study period (Figure 2a); (6) the Newfoundland and Labrador Climate Index (NLCI; Figure 2b), a mosaic of 10 environmental components that provides a measure of the overall state of environmental conditions and ecosystem variability in the NWA between 1951 and 2019 (Cyr & Galbraith, 2021). We note that data sources 1–4 represent observed data used for model fitting, while data sources 5 and 6 were used as input variables for the process model (Figure 3).

In the case of human removals, there were uncertainties in both the number of harvested animals and the age composition of harvests (animals less than 1 year old, or “young of the year” [YOY], vs. seals 1 year of age and older [referred to hereafter as adults]). These uncertainties were assessed and quantified based on previously published analyses (when possible) or expert opinion (see Appendix S1: Section S1). Not all animals that are harvested are successfully recovered and reported, a phenomenon typically referred to as “struck and loss.” The fraction of struck and loss varies by age class (YOY vs. adults), harvest source, and over time: accordingly, we assigned a time‐varying struck and loss rate for each harvest source based on previously published estimates (Hammill et al., 2015; Sjare & Stenson, 2002): for the Arctic and Greenland hunts, YOY struck and loss was set to 50% and for the Canadian hunt, it was set to 1% prior to 1983 and 5% from 1983 onward. The struck and loss rate for adults was set to 50% for all sources of harvest (Hammill et al., 2015). Removals from bycatch were assumed to be fully reported, so the struck and loss rate was set to 0% for this mortality source. For each human removal source, we defined variables *Q*_0,*t*_ and *Q*_*A,t*_ as the proportion of deaths recovered and reported (for YOY and adults, respectively) in year *t*, calculated as 1—the rate of struck and loss.

To calculate the IC index, annual ice cover information (1969–2019) for whelping patch locations (Figure 1) was extracted from the *Canadian Ice Service of Environment Canada* online repository of sea ice conditions (https://iceweb1.cis.ec.gc.ca/IceGraph/page1.xhtml?lang=en). Data were acquired from the Gulf of St Lawrence and southern Labrador ice charts for two periods (*p*): whelping (*p* = 1; defined for Gulf as week of 28 February, and for Front as week of 26 March) and post‐weaning fast (*p* = 2; defined for Gulf as week of 5 March, and for Front as week of 9 April). The IC index for each year (*t*) and breeding area (*a*) was calculated as the relative area of the whelping patch covered by first‐year ice, expressed as a scaled deviation from typical ice cover, and averaged for the two periods (*p*):

(1) $$IC_{a,t}=\frac{1}{p}\sum_{p}\left(\mathrm{IC}E_{a,t,p}-\mu_{a,p}\right)\times \sigma_{a,p}^{-1}$$

where ICE_*a,t,p*_ is the raw ice cover data and μ_*a,p*_ and σ_*a,p*_ represent (respectively) the mean and standard deviation in ICE for each area/period over reference years 1969–2000 (Figure 2a).

### Model overview

The methods for analyzing harp seal population dynamics can be described in three parts: (1) the process model, a series of equations that describe demographic transitions and predict dynamics in the variables of interest; (2) the data model, which describes how empirical datasets are related to the predicted dynamics of the process model; and (3) model fitting. We describe each of these steps in the following sections, and we provide a graphical schematic of model structure to help illustrate the functional relationships between elements of the model (Figure 3).

### Process model

#### Summary

Population dynamics are described using a female‐based, age‐structured matrix model with discrete annual time steps. The abundance of animals in each age class for a given year corresponds to the population state in late winter, that is, immediately before the annual pup survey. This formulation simplified interpretation of age samples and pregnancy rate data, as pup counts could be compared with expected births given the age structure and pregnancy rate of females sampled in the same year. The population vector **n**(*t*) describes the number of individuals in each age class, *i* (*i* = 1, 2… 36), at year *t*, and **N**(*t*) is the sum of **n**(*t*) across age classes. The first entry in the population vector (**n**(1,*t*)) corresponds to 1‐year olds: that is, pups born the previous year that have survived until their first birthday. The last age class, *i* = 36, represents a multi‐year class comprised of all animals older than 35 years of age. Although newborn YOY are not directly included in the population vector **n**(*t*), their numbers are separately tracked as the summed annual reproductive output of adult females. Demographic transitions between **n**(*t*) and **n**(*t + 1*) are calculated from annually varying vital rates: fecundity (*F*; the probability that a female gives birth at year *t*), YOY survival ($S_{0}$, the probability that a newborn survives to its first birthday), and adult survival ($S_{A}$).

#### Survival

We used a competing hazards formulation to model survival, which provides a mathematically coherent way to incorporate multiple hazards into a population analysis (e.g., Beyersmann et al., 2009; Brodie et al., 2013; Gelfand et al., 2000; Heisey & Patterson, 2006). In this formulation, a discreet set of effectively independent causes of death (hazards) are considered: During each time step, individuals are exposed to risk from all hazards (the temporal sequence of exposure to different hazards within a time step is not explicitly considered), but at any instant, mortality from one hazard precludes mortality from all other hazards. Annual survival rates for both YOY (S_0_) and adults (S_A_) thus represent the joint probability of surviving all competing hazards, and were calculated as:

(2) $$S_{0}\left(t\right)=\exp\left(-\left[h_{0}\left(t\right)+h_{\mathrm{IC}}\left(t\right)+h_{H0}\left(t\right)\right]\right)$$

(3) $$S_{A}\left(i,t\right)=\exp\left(-\left[h_{A}\left(i,t\right)+h_{\mathrm{HA}}\left(t\right)\right]\right)$$

The instantaneous hazard terms (*h*) in Equations (2) and (3) include natural hazards for YOY (*h*_0_) and adults (*h*_A_), hazards for YOY associated with poor ice conditions (*h*_IC_), and hazards associated with human removals of YOY (*h*_H0_) and adults (*h*_HA_). In the following sections, we describe calculations for each of these hazard terms, which we formulated in terms of log hazards (γ) to allow multiple predictor variables to be combined using simple additive equations.

##### Natural hazards

We define natural hazards to include all sources of mortality other than human removals and ice‐related mortality for YOY, which we modeled separately (see below). We decompose natural hazards into sub‐equations describing (1) the effects of age and (2) the effects of density dependence and environmental variation. We calculated age effects using a slightly modified formulation of a Siler (1979) competing risk model:

(4) $$\gamma_{A}\left(i\right)=\alpha_{1}-\alpha_{2}\times \left(i-1\right)+\alpha_{3}\times {\left(i-1\right)}^{2}$$

In Equation (4), $\alpha_{1}$ determines average hazards for a 1‐year‐old juvenile, $\alpha_{2}$ allows for a reduction in hazards with age early in life, and $\alpha_{3}$ allows for an increase in hazards with age late in life. For YOY, we assumed that natural log hazards ($\gamma_{0}$) are equivalent to those of a 1‐year‐old juvenile with an added risk component (υ):

(5) $$\gamma_{0}=\gamma_{A}\left(1\right)+\upsilon$$

We next incorporated the effects of density dependence and environmental factors. For long‐lived species such as harp seals, adult survival is typically buffered from effects of environmental variation and density dependence (Eberhardt, 2002). These effects were therefore expected to primarily impact YOY in their first year of life and were calculated as:

(6) $$\gamma_{D0}=\phi_{S}\times N\left(t\right)\times \exp\left(\delta \times \text{NLCI}\left(t\right)+\varepsilon_{S}\left(t\right)\right)$$

where parameter ϕ_*S*_ determines the *per capita* strength of density‐dependent effects under average conditions, δ determines the effect of environmental factors (as described by the NLCI), and random effect term ε_*S*_ accounts for additional stochasticity and is drawn as a random normal variate with mean of 0 and standard deviation σ_*S*_. Our formulation of Equation 6 assumes that the survival impacts of variable environmental conditions will increase as the population approaches carrying capacity, and thus these effects were included as an exponentiated modifier of the density‐dependent term following Stewart et al. (2025). When NLCI and ε_*S*_ are at their average values of 0, the exponentiated multiplier = 1, while deviations from average environmental conditions lead to increases or decreases in the strength of density‐dependent effects.

For older age classes, we assumed that the magnitude of effects of environmental variation and density dependence would be proportionally lower than for YOY:

(7) $$\gamma_{\mathrm{DA}}=\log\left(\left[\exp\left(\gamma_{D0}\right)-1\right]\times \zeta +1\right)$$

Equation (7) was formulated to facilitate interpretation of adjustment parameter ζ, which is constrained to the 0–1 scale: if ζ = 1, the *per capita* effects of density dependence and environmental variation on hazard rates are equivalent for YOY and adults; if ζ = 0.5, effects for adults (i.e., proportional increases in mortality) are half that for YOY; if ζ = 0 there are no density‐dependent or environmental effects on adult survival.

We summed the log‐hazard effects described in Equations ((4), (5), (6), (7))–((4), (5), (6), (7)) and transformed by exponentiation to derive the natural hazard terms for YOY (*h*_0_) and adults (*h*_A_):

(8) $$h_{0}\left(t\right)=\exp\left(\gamma_{0}+\gamma_{D0}\right)$$

(9) $$h_{A}\left(i,t\right)=\exp\left(\gamma_{A}\left(i\right)+\gamma_{\mathrm{DA}}\right)$$

##### Ice hazards

The log‐transformed hazards of ice‐related mortality for YOY in each breeding area, *a*, were calculated as a logit function of the ice anomaly index (IC):

(10) $$\gamma_{\mathrm{IC}}\left(a,t\right)=\text{logi}t^{-1}\left(-\left(\psi_{0}+\psi_{a}\times \mathrm{IC}\left(a,t\right)\right)\right)$$

where parameter ψ_0_ determines the range of ice anomaly values over which ice‐related mortality increases and ψ_a_ determines the magnitude of the effect of ice anomalies in area *a* on YOY mortality. This functional form was selected for consistency with previous research (Stenson & Hammill, 2014) suggesting that in years with average or above‐average ice cover there is little or no ice‐related mortality, but in years when the ice cover is significantly below average the mortality rate may increase sharply. To calculate the population‐level hazards from area‐specific ice anomalies, we transformed Equation (10) by exponentiation and took the weighted average across breeding areas, with weighting determined by the proportion of YOY born in each breeding area (*P*(*a,t*)):

(11) $$h_{\mathrm{IC}}\left(t\right)=\sum_{a=1}^{3}P\left(a,t\right)\times \exp\left(\gamma_{\mathrm{IC}}\left(a,t\right)\right)$$

For years in which pup surveys were conducted (*t*_s_), the values of *P*(*a,t*_s_) were calculated directly from survey data: for all other years, *P*(*a,t*) was drawn from a Dirichlet distribution with parameters θ_*a*_ calculated by pre‐fitting a Dirichlet distribution (using maximum likelihood methods) to the observed values of *P*(*a,t*_s_).

##### Human removal hazards

For YOY and adults, we calculated the annual hazards associated with each of the four sources of human mortality (*j*) as derived hierarchical parameters:

(12) $$\gamma_{H0,j}\left(t\right)\sim \text{normal}\left({\bar{\gamma }}_{H0,j},\sigma_{H,j}\right)$$

(13) $$\gamma_{\mathrm{HA},j}\left(t\right)\sim \text{normal}\left({\bar{\gamma }}_{\mathrm{HA},j},\sigma_{H,j}\right)$$

where the hyper‐parameters to be estimated include average source‐specific log‐hazards from human removals for YOY (${\bar{\gamma }}_{H0,j}$) and adults (${\bar{\gamma }}_{\mathrm{HA},j}$), and the magnitude of variance in hazards from year to year ($\sigma_{H,j}$). We then converted log hazards to hazard rates by exponentiation:

(14) $$h_{H0,j}\left(t\right)=\exp\left(\gamma_{H0,j}\left(t\right)\right)$$

(15) $$h_{\mathrm{HA},j}\left(t\right)=\exp\left(\gamma_{\mathrm{HA},j}\left(t\right)\right)$$

#### Fecundity

Fecundity was calculated using an instantaneous hazard function (the hazard of not becoming pregnant) for consistency with survival rate calculations. Pregnancy rates for the first 3 age classes are negligible (Stenson, Buren, & Sheppard, 2020), and consequently, we set fecundity to 0 for *i* < 3 following Hammill et al. (2015). For older females, we calculated fecundity as:

(16) $$F\left(i,t\right)=\begin{array}{c} \left\{\begin{array}{cc} \exp\left[-\exp\left(\beta_{1}+\beta_{2}\times {\left(8-i\right)}^{2}+\phi_{F}\times N\left(t-1\right)\times \nabla \right)\right] & \text{if}\;4\leq i\leq 8\\ \exp\left[-\exp\left(\beta_{1}+\phi_{F}\times N\left(t-1\right)\times \nabla \right)\right] & \text{if}\;i>8 \end{array},\right.\\ \;\text{and}\;\nabla =\exp\left\{\delta \times \text{NLCI}\left(t-1\right)+\varepsilon_{F}\left(t\right)\right\} \end{array}$$

In Equation (16), parameter β_1_ determines the average pregnancy rate in the absence of other effects, and the additive terms to the right of β_1_ can be interpreted as log hazard ratios, decreasing (in the case of terms >0) or increasing (terms <0) the probability of pregnancy. Parameter β_2_ determines the effect of age for younger adults (pregnancy rates are assumed to reach an asymptote by age 8; Hammill et al., 2015), ϕ_F_ determines the strength of density‐dependent effects, δ determines the effect of environmental conditions assuming a 1‐year lag (i.e., female fecundity in year *t* is primarily affected by the NLCI value in year *t*−1), while ε_*F*_ is a year‐specific random effect capturing annual variation in fecundity that affects all age classes, and was drawn from a normal distribution with mean of 0 and standard deviation σ_*F*_. As was the case for survival, our formulation of Equation (16) assumed that the impact of variable environmental conditions on fecundity would increase as the population approaches carrying capacity, and these effects were therefore incorporated as an exponentiated modifier of the density‐dependent term.

#### Estimated population dynamics

Vital rates were organized into an age‐structured and time‐varying projection matrix, **M**(*t*), (Caswell, 2001) of dimensions 36 × 36, which describes all demographic transitions at year *t*:

(17) $$M\left(t\right)=\left[\begin{array}{ccccccc} R\left(1,t\right) & R\left(2,t\right) & R\left(3,t\right) & R\left(4,t\right) & \cdots & R\left(35,t\right) & R\left(36,t\right)\\ S_{A}\left(1,t\right) & 0 & 0 & 0 & \cdots & 0 & 0\\ 0 & S_{A}\left(2,t\right) & 0 & 0 & \cdots & 0 & 0\\ 0 & 0 & S_{A}\left(3,t\right) & 0 & \cdots & 0 & 0\\ 0 & 0 & 0 & S_{A}\left(4,t\right) & \cdots & 0 & 0\\ \vdots & \vdots & \vdots & \vdots & \ddots & \vdots & \vdots \\ 0 & 0 & 0 & 0 & \cdots & S_{A}\left(35,t\right) & S_{A}\left(36,t\right) \end{array}\right]$$

In Equation (17), the terms *R*(*i,t*) in the first row of the matrix represent reproductive contributions of females to the 1‐year‐old age class the following year, and were calculated as the product of age‐specific fecundity and YOY survival, adjusted for a 50:50 sex ratio:

(18) $$R\left(i,t\right)=0.5\times F\left(i,t\right)\times S_{0}\left(t\right)$$

We calculated the expected population vector in the following year, **n**(*t + 1*), via matrix multiplication:

(19) $$n\left(t+1\right)=M\left(t\right)\times n\left(t\right)$$

We note that our computation of population dynamics by matrix projection does not explicitly account for demographic stochasticity; however, given the magnitude of abundance (in the millions), we expected that the impacts of demographic stochasticity would be negligible relative to the impacts of environmental stochasticity and thus could be safely ignored. To initiate the population vector at *t* = 0 we multiplied the starting abundance ($N_{0}$, a parameter to be estimated) by the stationary age distribution (SAD) associated with the demographic schedule for *t* = 1. The *SAD* was calculated during model fitting by projecting the **M**(t) matrix forward in time until the stage distribution stabilized.

#### Model‐predicted variables

We differentiate model‐predicted, “observable” variables (derived from latent variables in the process model; Figure 3) from their corresponding observed data variables, and for clarity, we identify the former using the superscript “prd,” and the latter using the superscript “obs.” The model‐predicted number of YOY available for counting during a survey in year *t* was calculated as:

(20) $$\begin{array}{c} \mathrm{YO}Y^{\mathrm{prd}}\left(t\right)=0.5\times \left(\sum_{i=1}^{36}n\left(i,t\right)\times F\left(i,t\right)\right)\times S_{\mathrm{nb}}\left(t\right),\\ S_{\mathrm{nb}}\left(t\right)={\left(S_{0}\left(t\right)\right)}^{\pi \left(t\right)} \end{array}$$

Equation (20) assumes a 50:50 sex ratio and adjusts the total number of pups born for newborn survival, *S*_nb_(*t*), which accounts for the fact that a small fraction of YOY mortality can occur prior to the pup survey (i.e., not all pups born are available to be counted). The fraction of first year mortality occurring before the survey was determined by annually varying parameter π(*t*), which was constrained to the 0–1 scale.

The predicted age frequency distribution at year *t*, Agedist^prd^(*t*), was calculated as a linear array:

(21) $$\begin{array}{l} \text{Agedis}t^{\mathrm{prd}}\left(t\right)=\left[n\left(m,t\right),n\left(m+1,t\right),\ldots n\left(36,t\right)\right]\\ \;\times {\left[\sum_{i=m}^{36}n\left(i,t\right)\right]}^{-1} \end{array}$$

where *m* is the minimum age of adults to be considered for comparison with observed age distributions. We set *m* = 5 years to avoid potential biases associated with collections of younger animals, and for consistency with previous analyses (Tinker et al., 2023).

The predicted number of reported human removals for YOY from source *j* was calculated in two steps: first, we calculated the expected fraction of all YOY deaths accounted for by source *j* in year *t*:

(22) $$f_{H0,j}\left(t\right)=h_{H0,j}\left(t\right)\times {\left[h_{0}\left(t\right)+h_{\mathrm{IC}}\left(t\right)+\sum_{j}h_{H0,j}\left(t\right)\right]}^{-1}$$

We note that Equation (22) reflects the additive nature of competing instantaneous hazards, providing a straightforward estimator for the expected proportion of deaths attributable to cause *j* within a time step (Heisey & Patterson, 2006; Joly et al., 2009), independent of their order of occurrence. Next, this fraction was used to calculate the predicted reported removals of YOY from source *j* in year *t*:

(23) $$\begin{array}{l} Hv_{0,j}^{\mathrm{prd}}\left(t\right)=\left[0.5\times \left(\sum_{i=1}^{36}n\left(i,t\right)\times F\left(i,t\right)\right)\right.\\ \;\left.\times \left(1-S_{0}\left(t\right)\right)\times f_{H0,j}\left(t\right)\times Q_{0,j}\left(t\right)\right] \end{array}$$

where the term enclosed by square brackets gives the total expected deaths for YOY over the year, and $Q_{0,j}\left(t\right)$ represents the expected proportion of removals of YOY from source *j* that would be recovered and reported (i.e., inverse of struck‐and‐lost) in year *t*.

Similarly, the expected fraction of total adult deaths in each age class attributable to human removals from source *j* in year *t* was calculated as:

(24) $$f_{\mathrm{HA},j}\left(i,t\right)=h_{\mathrm{HA},j}\left(t\right)\times {\left[h_{A}\left(i,t\right)+\sum_{j}h_{\mathrm{HA},j}\left(t\right)\right]}^{-1}$$

We note that Equation (24) implicitly assumes that human removals for the adult age classes are proportional to the current age structure at time of harvest. The resulting fraction was used to calculate the predicted reported removals of adults in year *t* from source *j*:

(25) $$Hv_{A,j}^{\mathrm{prd}}\left(t\right)=\sum_{i=1}^{36}\left[n\left(i,t\right)\times \left(1-S_{A}\left(i,t\right)\right)\right]\times f_{\mathrm{HA},j}\left(i,t\right)\times Q_{A,j}\left(t\right)$$

where the term enclosed by square brackets gives the total expected deaths for each age class and $Q_{A,j}\left(t\right)$ is the proportion of removals of adults from source *j* that were recovered and reported in year *t*. The total expected annual reported removals from each source, ${\mathrm{Hv}}_{j}^{\mathrm{prd}}\left(t\right)$, was calculated as the sum of ${\mathrm{Hv}}_{0,j}^{\mathrm{prd}}\left(t\right)$ and ${\mathrm{Hv}}_{A,j}^{\mathrm{prd}}\left(t\right)$.

### Data model

The observed YOY abundance estimates (YOY^obs^; Appendix S1: Table S1) at time *t* were assumed to follow a gamma distribution:

(26) $$\mathrm{YO}Y^{\mathrm{obs}}\left(t\right)\sim \text{gamma}\left(a=\frac{{\left[\mathrm{YO}Y^{\mathrm{prd}}\left(t\right)\right]}^{2}}{{\left[SE_{\mathrm{sv}}\left(t\right)\right]}^{2}},b=\frac{\mathrm{YO}Y^{\mathrm{prd}}\left(t\right)}{{\left[SE_{\mathrm{sv}}\left(t\right)\right]}^{2}}\right)$$

where the error estimates associated with each YOY survey point estimate ($SE_{\mathrm{sv}}$) were computed prior to model fitting according to the survey design methods (Appendix S1: Table S1; Stenson et al., 2022) and provided to the model as input data.

Data on pregnancy rates of sampled females (Appendix S1: Table S2) were treated as a beta‐binomial variable: specifically, given a sample of females of age *i* in year *t* (NF(*i, t*)), the observed number of pregnant females (NPr^obs^(*i, t*)) was assumed to follow a beta‐binomial distribution with probability determined by the model‐predicted fecundity rates:

(27) $$\mathrm{NP}r^{\mathrm{obs}}\left(i,t\right)\sim \text{beta}.\text{binomial}\left(\mathrm{NF}\left(i,t\right),\eta \times F\left(i,t\right),\eta \times \left[1-F\left(i,t\right)\right]\right)$$

where estimated inverse scale parameter η determines the magnitude of variability in sampled pregnancy rates over and above what would be expected from a simple binomial distribution.

Counts of adult animals of age *i* sampled in year *t*(NC(i,*t*); Appendix S1: Table S3) were compiled into linear arrays for each year, Agedist^obs^(*t*) = [NC(*m*,*t*), NC(*m* + 1,*t*)… NC(*36,t*)], where *m* = 5 was the minimum age to be considered for model fitting (Tinker et al., 2023). The observed age distributions were compared to the model‐predicted age distribution (Agedist^prd^(*t*)), and to account for measurement uncertainty and sampling error in the age counts, we used a Dirichlet‐multinomial formulation:

(28) $$\text{Agedis}t^{\mathrm{obs}}\left(t\right)\sim \text{Dirichlet}.\text{multinomial}\left(\sum_{i=m:36}\mathrm{NC}\left(i,t\right),\tau \times \text{Agedis}t^{\mathrm{prd}}\left(t\right)\right)$$

where τ is an estimated precision parameter.

The annual reported human removals from source *j* (Appendix S1: Table S4) were assumed to follow negative‐binomial distributions with mean corresponding to the model‐predicted values:

(29) $$Hv_{j}^{\mathrm{obs}}\left(t\right)\sim \text{negative}.\text{binomial}\left(\bar{x}=Hv_{j}^{\mathrm{prd}}\left(t\right),\xi_{j}\left(t\right)\right)$$

In Equation (29), the annually varying precision parameter (ξ) was calculated from the previously assigned coefficient of variation (CV) associated with each removal source and time period (Appendix S1: Table S5):

(30) $$\xi_{j}\left(t\right)={\left(Hv_{j}^{\mathrm{prd}}\left(t\right)\right)}^{2}\times {\left[{\left(CV_{j}\left(t\right)\times Hv_{j}^{\mathrm{prd}}\left(t\right)\left(t\right)\right)}^{2}-Hv_{j}^{\mathrm{prd}}\left(t\right)\right]}^{-1}$$

Next, to account for varying levels of uncertainty in the reported age composition of removal numbers from each source (i.e., YOY vs. adults), we treated reported numbers of harvested or by‐caught YOY (Appendix S1: Table S4) as draws from a beta‐binomial distribution:

(31) $$Hv_{0,j}^{\mathrm{obs}}\left(t\right)\sim \text{beta}.\text{binomial}\left(Hv_{j}^{\mathrm{obs}}\left(t\right),\kappa_{j}\left(t\right)\times \frac{Hv_{0,j}^{\mathrm{prd}}\left(t\right)}{Hv_{j}^{\mathrm{prd}}\left(t\right)},\kappa_{j}\left(t\right)\times \left[1-\frac{Hv_{0,j}^{\mathrm{prd}}\left(t\right)}{Hv_{j}^{\mathrm{prd}}\left(t\right)}\right]\right)$$

In Equation (31), the precision parameter κ_*j*_(*t*) was set for each source and year based on previously assigned levels of uncertainty associated with reported age compositions (Appendix S1: Table S5).

### Model fitting

Posterior distributions for parameters were estimated using standard Markov chain Monte Carlo (MCMC) methods. We used vague prior distributions for most parameters (i.e., scaled based on biological feasibility but having no information specific to the analysis). The Cauchy distribution (and half‐Cauchy for parameters logically constrained to be >0) is an effective uninformative prior because it is leptokurtic (“fat tailed”) and has no defined mean, and thus provides wide potential bounds on parameter space, a tendency to shrink towards 0 for non‐significant parameters, and minimized influence of the prior on the estimation of the posterior (Gelman, 2006; Gelman et al., 2008). A Cauchy prior was used for unbounded parameter δ and half‐Cauchy priors were used for $N_{0}$, η, τ, all variance parameters (σ), and all log‐hazard parameters (α, β, ϕ, γ), as these were constrained to be positive due to our inclusion of scaling constants in log‐hazard equations (Appendix S1: Section S1). For the remaining parameters (ψ, ζ, π, and υ), we set moderately informative priors based on published information and/or biological feasibility, and to improve model identifiability (Appendix S1: Section S1 and Table S6).

We used R (R.Core.Team, 2022) and Stan software (Carpenter et al., 2017) to code and fit the model, saving 20,000 samples after a burn‐in of 1000 samples. We evaluated model convergence by graphical examination of trace plots from 10 independent chains and by ensuring that Gelman–Rubin convergence diagnostic (R‐hat) was <1.1 for all fitted parameters. We plotted and visually compared prior and posterior distributions for all parameters to assess the degree to which posteriors were distinct from priors. We conducted graphical posterior predictive checking to evaluate model goodness of fit, ensuring that out‐of‐sample predictive distributions of YOY abundance, female pregnancy rates, and age distributions were consistent with distributions of observed data. We also calculated the posterior predictive Bayesian *p* values for each dataset (using summed Pearson residuals as the test statistic to compare observed vs. out‐of‐sample predicted data), with reliable model fit indicated by 0.05 < Bayesian *p* < 0.95. We employed leave‐out‐one cross‐validation approximation (R library “Loo”; Vehtari et al., 2017) to estimate the expected log predictive density (ELPD) for a new dataset and used the Pareto k diagnostic to identify data observations exerting excessive influence on the joint posterior (we define excessive influence as Pareto‐k > 1). As an additional goodness‐of‐fit check, we created “out‐of‐sample” hindcast projections of population dynamics to compare to the fitted model projections. Specifically, for each out‐of‐sample projection, we initiated a “new” 1951 population at $N_{0}$, and then projected forward for *T* years with the process model parameterized by drawing from the joint posteriors of all parameters and with hierarchical random effects drawn from their appropriate sampling distributions (except for hierarchical harvest hazard parameters, which were set to their mean estimated values). In the case of a well‐fit model, the best‐fit abundance trend should fall within the distribution of out‐of‐sample hindcast projections. Model results were summarized by reporting the mean and 95% Credible Interval (CI) of the posterior distributions for base model parameters and derived parameters.

### Sensitivity analyses

We used samples from the joint posterior to parameterize a Bayesian life stage simulation analysis (LSA; Eacker et al., 2017, Wisdom et al., 2000), which we used to (1) assess the sensitivity of population growth rate (λ) to proportional perturbations in cause‐specific hazard rates and fecundity (we back‐transformed fecundity to instantaneous hazard units for comparison to survival hazards); and (2) evaluate the degree to which variation in each hazard rate contributed to variation in λ over three time periods: 1951–1982, 1983–1999, and 2000–2019. Methods for the LSA were based on those described by Eacker et al. (2017) and are described in detail in Appendix S1: Section S1. We also graphically evaluated trends in the magnitude of individual mortality sources for YOY and adults over the study period.

## RESULTS

Model fitting resulted in well‐mixed chains, with R‐hat <1.1 for all parameters (Appendix S1: Table S7). Graphical posterior predictive checks showed consistency between observed and out‐of‐sample distributions for all datasets (Appendix S1: Figure S1), although the Bayesian P value for YOY surveys (0.975) indicated marginal goodness of fit for this particular dataset. Leave‐out‐one cross‐validation analysis revealed the cause of marginal goodness of fit, as three YOY survey data points (1990, 1994, 2008) had Pareto *k* values >1 and thus exerted excessive leverage on the overall model likelihood. This outcome was not surprising given that the observer error for this dataset was pre‐computed rather than estimated by the model (as for reproductive rates and age composition data). Refitting the model with these three data points excluded resolved the goodness‐of‐fit issue (new Bayesian P for YOY surveys = 0.688, all Pareto‐*k* values <1) and had minimal impacts on other model parameters; however, because these survey results contributed important information about pup abundance during periods of rapid change, we decided to retain all data points and we report all further results for the initial model fit. The distribution of out‐of‐sample hind‐cast projections of population trends were consistent with the best‐fit projection (Appendix S1: Figure S2), and posterior distributions were distinct from prior distributions for all parameters except those with moderately informative priors (Appendix S1: Figure S3). Detailed statistics for all model parameters are provided in Appendix S1: Table S7.

**FIGURE 4:**
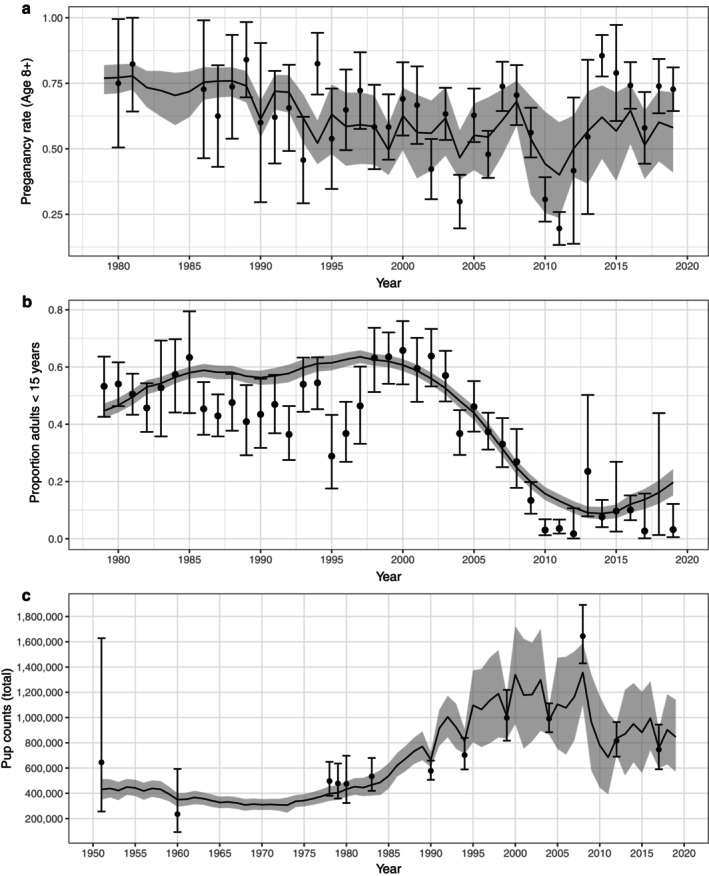
Model estimates of temporal trends in pregnancy rates, age composition, and pup production. Mean posterior estimates are plotted as solid black lines, 95% CI plotted as shaded gray bands, and observed data are overlain as points with error bars showing the 95% confidence interval around each sample point, calculated from survey design‐based standard error estimates. (a) Estimated pregnancy rates for females 8 yrs. or older, 1978–2020; (b) estimated proportion of younger age classes in the population (age between 8 and 15 years), 1978–2020; (c) estimates of temporal variation in pup production, 1950–2019.

The model projections for each data series were consistent with observed raw datasets, effectively describing variation in observed adult pregnancy rates (Figure 4a), relative age structure (Figure 4b), and pup abundance (Figure 4c). The pup survey estimates for 1990, 1994 and 2008 deviated most from expected pup trends, and indeed were the data points with elevated Pareto k estimates. Estimated pup production in 1951 was 430,000 (95% CI = 350,000–512,000), declining to a minimum of 308,000 (95% CI = 262,000–355,000) in 1968, then increasing to a maximum of 1.36 million (95% CI = 1,102,000–1,588,000) in 2008 (Figure 4c). Pup production declined again to 685,000 (95% CI = 392,000–1,037,000) by 2011, but has since recovered to an estimated 846,000 (95% CI = 572,000–1,141,000) in 2019.

**FIGURE 5:**
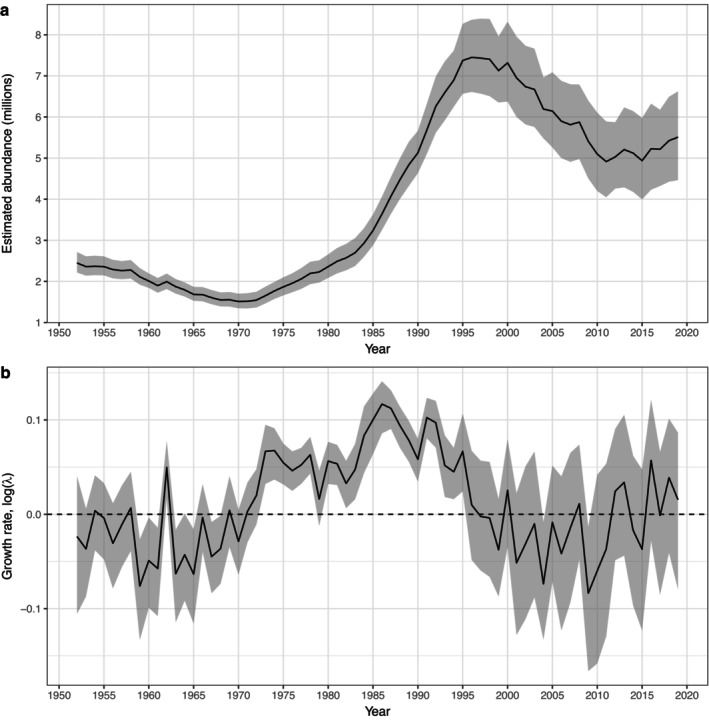
Model estimates of temporal variation in population abundance and growth rates, from 1950 to 2019. Mean posterior estimates are plotted as a solid black line, and 95% CI are plotted as a shaded gray band. (a) Estimated trends in abundance, representing the sum of all adult age classes and young of the year; (b) estimated trends in population growth, presented as log‐transformed values of λ, the annual finite growth rate.

Estimated total abundance trends represent a latent parameter in the model, having no corresponding observed dataset (Figure 5a). Estimated trends varied considerably over the study period: the estimated abundance in 1951 was 2.4 million (95% CI = 2.2–2.7 million), and the population then declined to a minimum of 1.5 million (95% CI = 1.3–1.7 million) in 1970, reflecting a mean intrinsic growth rate (where *r* = log(λ)) of $\bar{r}$ = −0.027 (Figure 5b). After 1970, the population increased rapidly to a maximum of 7.4 million (95% CI = 6.5–8.4 million) in 1997 (Figure 5a), reflecting a mean growth rate of $\bar{r}$ = 0.059 over this period (Figure 5b). Estimated abundance began to decline again after 1997 with a mean growth rate of $\bar{r}$ = −0.030, reaching a low of 4.9 million (95% CI = 4.1–5.9 million) in 2011 (Figure 5a,b), but then stabilized and increased slightly to 5.5 million (95% CI: 4.5–6.6 million) by 2019.

**FIGURE 6:**
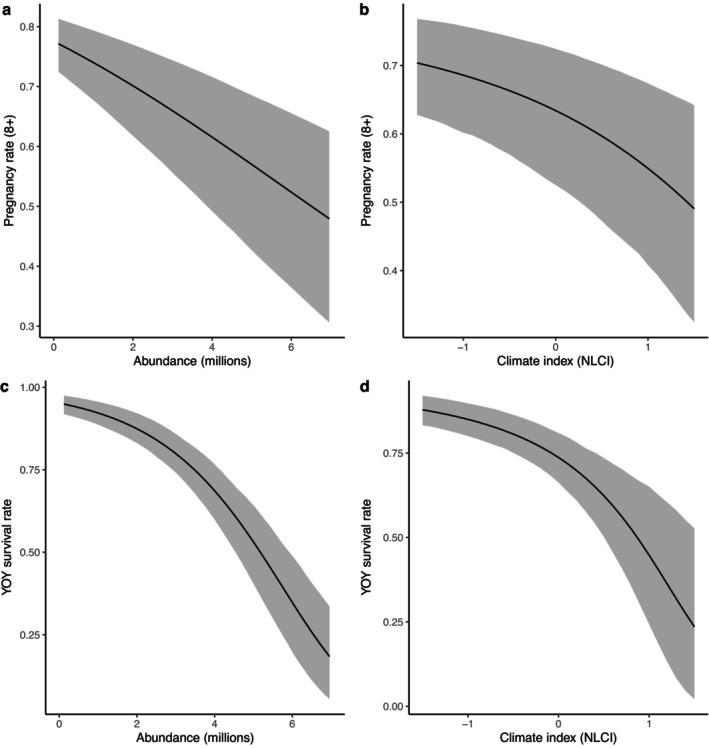
Model‐based estimates of the effect of population abundance (a, c) and the Newfoundland and Labrador Climate Index (b, d) on pregnancy rates (a, b) and young‐of‐the‐year (YOY) survival (c, d). Solid black lines indicate mean estimated values and gray shaded bands indicate the associated 95% CI.

**FIGURE 7:**
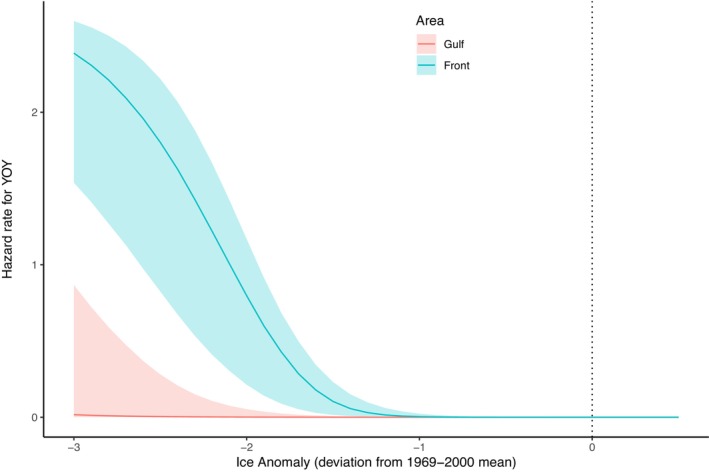
Changes in model‐estimated contribution of ice anomaly hazards (presented as instantaneous hazard rates) to young of the year mortality. More negative ice anomaly values represent years with less ice. The effects of ice index are estimated separately for the Gulf and the Front. Solid lines indicate mean estimated values and shaded bands indicate the associated 95% CI.

Both pregnancy rates and YOY survival varied significantly with density, decreasing as population abundance increased (Figure 6a,b). Pregnancy rates and YOY survival were also negatively correlated with the NLCI (Figure 6c,d). Years with anomalously low ice cover in the Gulf of St. Lawrence and the Front were associated with increases in YOY mortality, although ice effects appeared to be most significant for the Front breeding patch (Figure 7). Survival rates also varied with age, showing the inverted‐U pattern typical of mammalian survivorship schedules (Caughley, 1966) with density‐dependent impacts mostly affecting YOY, juveniles, and very old individuals (Appendix S1: Figure S4).

**FIGURE 8:**
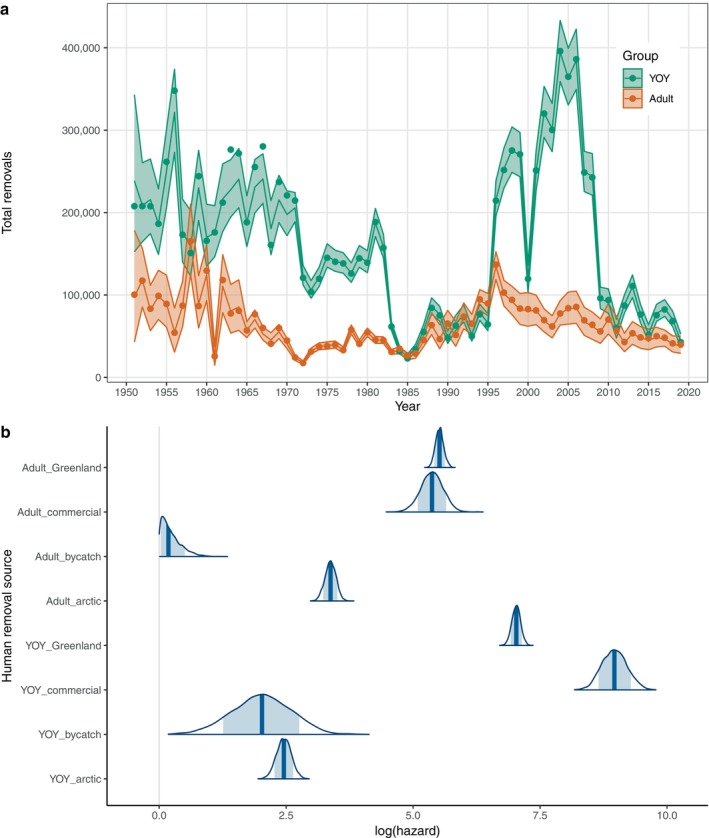
Trends in model‐estimated mortality from human removals. (a) Temporal variation in total removals: Mean estimates are plotted as solid lines with associated 95% CI plotted as shaded bands. Observed data on reported deaths are plotted as points for comparison (model estimates have been adjusted for struck and loss to be comparable to reported deaths). (b) Estimated posterior distributions for log‐hazard rates, plotted by mortality source and age class. Point estimates are shown as solid vertical lines, and 80% CI are indicated as shaded areas under each density curve.

The model successfully captured temporal trends in total removals, which were dominated by YOY in most years (Figure 8a). The four sources of human removal differed dramatically in terms of their per capita hazard rates and the relative magnitude of hazards for YOY versus older age classes (Figure 8b). The Canadian commercial harvest and Greenland harvest had substantially higher mean hazard rates than the Arctic hunt or bycatch mortality. YOY hazards were highest for Canadian commercial harvests, while adult hazards were highest for Greenland harvests. Similarly, hazard rates associated with the Arctic hunt were higher for adults than YOY, while bycatch hazards were greater for YOY than for adults (Figure 8b).

**FIGURE 9:**
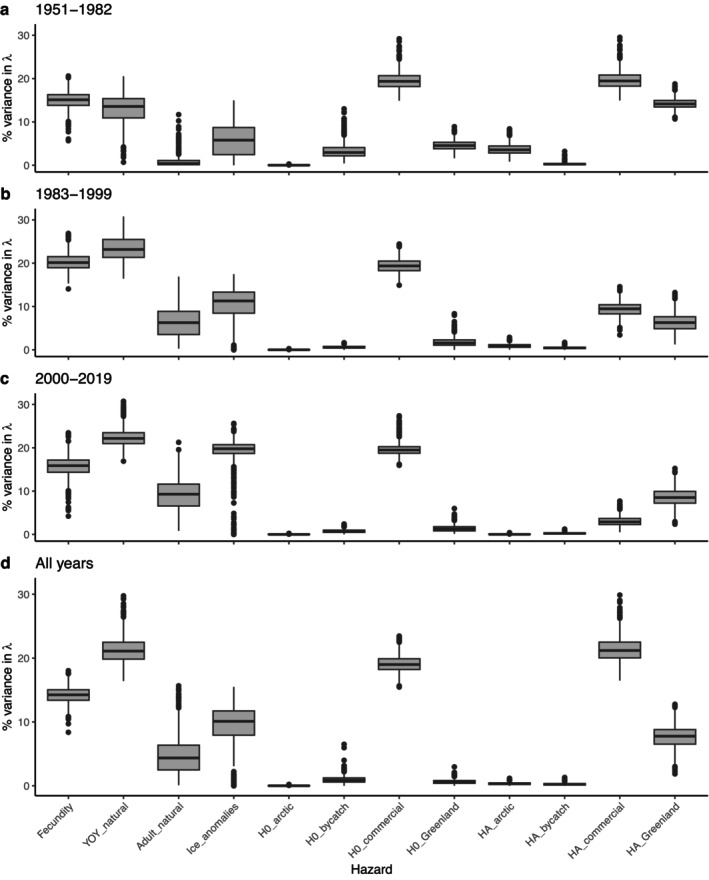
Results from Bayesian life stage analysis, comparing the relative degree to which variation in instantaneous hazard rates for fecundity and cause‐specific sources of mortality (for young of the year and adults) contributed to variation in annual growth rates (λ) over different time periods: (a) 1951–1982; (b) 1983–1999; (c) 2000–2019; (d) the entire study period (1951–2019).

The results of the Bayesian LSA showed variable impacts on population growth (λ) of age‐ and cause‐specific hazards. While asymptotic lambda estimates generated by a Bayesian LSA (λ_sim_) can potentially differ from realized population growth rates (λ_obs_) in a time‐variant environment, in the current analysis we found that the distributions of λ_sim_ values for each period were reflective of (and did not differ significantly from) the corresponding distributions of λ_obs_ values (Appendix S1: Figure S6), providing confidence in our LSA results. For a given magnitude of perturbation, the hazards with the strongest effects on λ were adult natural hazards and human removal hazards for adults (*h*_A_ and *h*_HA_), while the relative effects of YOY natural hazards (*h*_0_), ice anomaly hazards (*h*_IC_), YOY human removal hazards (*h*_H0_), and fecundity (*F*) were considerably lower (Appendix S1: Figure S5). These results are consistent with typical age‐based patterns of vital rate elasticities for long‐lived vertebrates (Caswell, 2001), but do not reflect the realized contributions of different hazards to historical variation in λ, which depend not only on elasticities but also on the actual patterns of variation in each rate (Wisdom et al., 2000). A comparison of the proportional contributions of hazards to observed variation in λ provided a clearer picture of the relative importance of age‐ and cause‐specific hazards in this system (Figure 9). In the first three decades of the study period (1951–1982), the strongest driver of variation in λ was the Canadian commercial harvest, both for YOY and for adults, although the adult Greenland harvest, fecundity, and YOY natural hazards also had substantial effects (Figure 9a). Over the following two decades (1983–1999), a period of mostly positive growth (Figure 5b), the relative contribution of the adult commercial harvest declined, while the contributions of ice anomaly hazards and natural hazards for YOY and adults increased (Figure 9b). In the most recent two decades (2000–2019), the contributions of the commercial harvest hazards for adults further declined and were surpassed by the effects of the adult Greenland harvest, while the relative contributions of ice anomaly hazards and natural hazards for YOY and adults continued to increase (Figure 9c). Considering the entire study period, our results indicate that most of the variation in λ has been explained by variation in natural hazards for YOY and commercial harvest hazards for YOY and adults, with slightly lower contributions from hazards associated with fecundity, ice anomalies, and the adult Greenland harvest (Figure 9d).

**FIGURE 10:**
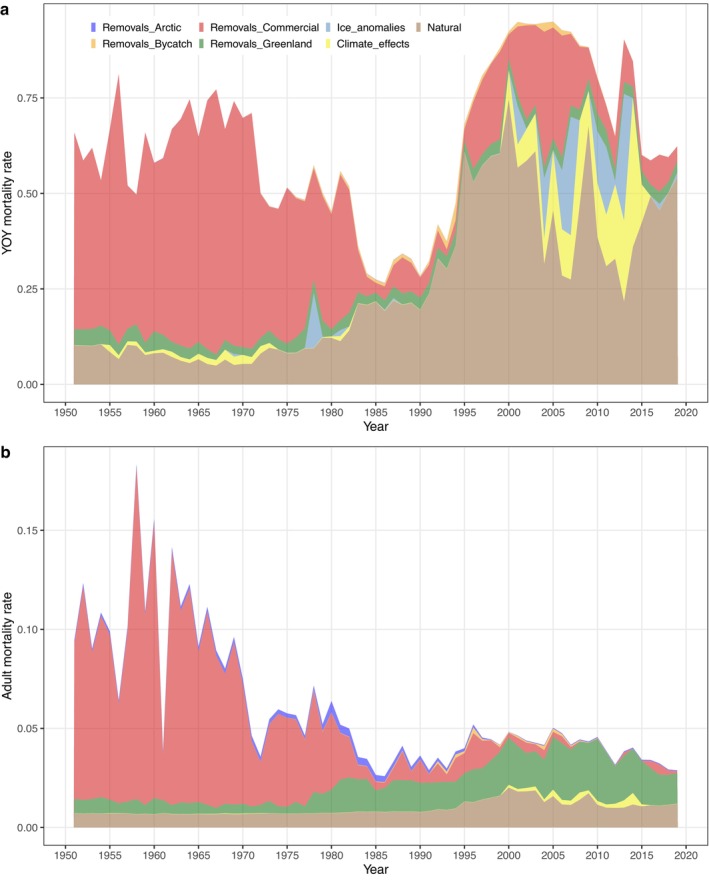
Plot showing temporal variation (1951–2019) in the relative contributions of various hazards to the total estimated mortality rate. Competing hazards include human removals from harvest or bycatch (partitioned by four sources), mortality attributable to anomalously poor ice conditions, excess mortality due to climate effects (as measured by NLCI), and all other natural hazards (which include density‐dependent mortality and stochastic variation). (a) Estimated hazard trends for young of the year. (b) Estimated hazard trends for adults. To account for age‐based differences in mortality, we have plotted here the weighted average of hazard rates across all adult age classes, with weights corresponding to the average stationary age distribution (i.e., the relative number of individuals in each year class).

We also graphically evaluated how the magnitude of different mortality sources has varied over time. In the case of YOY, human removals (dominated by the Canadian commercial harvest) represented the largest component of mortality up until 1983 (Figure 10a). Harvest hazards dropped sharply between 1985 and 1995, leading to greatly reduced YOY mortality overall. By 1995, an increase in abundance resulted in an uptick in natural mortality for YOY (Figure 10a), due primarily to density‐dependent effects. From 1995 to 2008, harvest mortality again increased, while mortality attributable to environmental factors and poor ice‐conditions was elevated from 2000 to 2015 (Figure 10a). More favorable environmental and ice conditions after 2015, combined with reduced human‐caused mortality, resulted in a slight reduction in total mortality at the end of the time series (Figure 10a).

The trends in adult hazards (Figure 10b) displayed not only some similarities with YOY hazards but also some key differences. As with YOY, the Canadian commercial harvest was the dominant contributor to mortality during the early years of the time series (1951–1983; Figure 10b). However, after 1983 the impact of harvest on adult mortality dropped sharply, and mortality from the Greenland hunt became the largest contributor to adult mortality for the rest of the time series (Figure 10b). There was a slight increase in natural hazards after 1995, reflecting density‐dependent effects; however, natural hazards remained relatively minor compared to mortality from human removals. Adult hazards from bycatch mortality, the Arctic hunt, and environmental effects have been minimal over the entire study period (Figure 10b).

## DISCUSSION

The results presented here demonstrate the potential of IPMs to help in inferring the drivers of trends in wildlife populations. The competing hazards formulation of survival enabled the partitioning of mortality into multiple discreet causes and, combined with the results of the simulation‐based sensitivity analysis, allowed us to assess the main drivers of harp seal mortality and population dynamics across seven decades in the NWA. We show that the NWA harp seal population, especially the juvenile component (YOY), faces multiple threats, but the relative influence of each of those threats has varied over time. Our model builds on the examples provided by other IPMs, which have explored mechanistic relationships between vital rates and environmental and anthropogenic processes (e.g., Eacker et al., 2017; Ersalman et al., 2025; Riecke, Lohman, et al., 2022; Riecke, Sedinger, et al., 2022). The results of our harp seal IPM demonstrate strong functional relationships between YOY survival and human removals (Figure 10a), environmental conditions (as represented by the NLCI index; Figure 6d), ice cover anomalies (Figure 7), and density‐dependent factors (Figure 6b). The latter relationship presumably reflects bottom‐up feedback processes such as intraspecific competition for prey resources (Solomon, 1958). Environmental conditions and density‐dependent processes also affect female pregnancy rates (Figure 6a, c). In contrast, adult survival rates were largely buffered from environmental conditions and density‐dependent processes, but were significantly impacted by human removals (Figures 9d and 10b).

The NWA harp seal population has shown dramatic swings in abundance over the past 70 years (Figure 5), and our results indicate that changes in the intensity of human removals—particularly the Canadian commercial harvest—are responsible for much of this volatility (Figure 9d). Prior to the early 1970s, high levels of harvest removals were the predominant contributors to both YOY and adult mortality (Figure 10) and thus the primary driver of population trends (Figure 9a). The resulting annual mortality rates (50% per year for YOY and 5%–10% per year for adults) led to a decline in the population, with abundance reaching a minimum by 1971. At that time, harvest quotas were implemented (Sergeant, 1991), leading to a reduction in both YOY and adult removals, and the population began to increase (Figure 5). Following the closure of the primary markets for whitecoats in 1983 (Sergeant, 1991), YOY harvests declined further (Figure 8). Overall, natural mortality remained low and the reduced harvest levels caused a sharp drop in overall mortality rates for both YOY and adults (Figure 10), permitting rapid population growth through the mid‐1990s (Figure 5a,b). The trends in abundance leveled off after 1995 and then began to decline, reflecting both a resumption of YOY harvests (Figure 8) as well as concurrent increases in density‐dependent mortality (natural hazards) and mortality associated with climate effects and poor ice conditions (Figure 10a). These combined factors led to a dramatic and unprecedented increase in YOY mortality, even surpassing levels seen in the 1970s. However, unlike the earlier period where mortality was primarily driven by harvest, recent mortality was more evenly split between increases in harvesting and higher natural mortality and ice‐related hazards. YOY mortality from these three hazards has been the main driver of population trends since 2000 (Figure 9c) and in addition to the decline in numbers (Figure 5) has resulted in a sustained shift in the age structure (Figure 3b).

Some of the recent increases in natural mortality, including the growing impacts of climate variables (Figure 10a), correspond to large‐scale, decadal shifts in the NWA ecosystem. The NWA food web is best described as an ecological wasp–waist pattern in which the crucial intermediate trophic link between zooplankton and large vertebrates is dominated by a single species, capelin (*Mallotus villosus*) (Buren et al., 2014, 2019). Capelin are a major prey species for many upper level predators such as piscivore fish, including Atlantic cod (*Gadus morhua*), sea birds, cetaceans, and harp seals (Buren et al., 2014; Beck et al., 1993; Lawson & Stenson, 1997; Sergeant 1991). A regime shift in the early 1990s associated with a cold‐water anomaly in the waters off Newfoundland and Labrador coincided with a collapse of the capelin stock off Newfoundland, as well as many groundfish stocks including Atlantic cod (Buren et al., 2014, 2019; Koen‐Alonso et al., 2021). Initially, the cold water was beneficial to harp seals as ice conditions improved. However, by 2000, both bottom temperatures and overall climate had largely returned to their prior state; yet, fish stocks did not substantially recover (Pedersen et al., 2017). Moreover, ice conditions began to deteriorate after 2000, while capelin stocks showed no signs of improvement, and other key prey for harp seal, such as Arctic cod (*Boreogadus saida*), which inhabit cold water, also declined in areas around Newfoundland (Stenson, Haug, & Hammill, 2020). The collapse in capelin abundance, along with changes in mid‐winter ice conditions, impacted YOY survival and female productivity (Stenson et al., 2016; Stenson, Buren, & Sheppard, 2020), both of which have contributed substantially to trends over the last two decades (Figure 9c).

The Newfoundland Climate Index (NLCI) that we used as a climate variable in our model was developed to represent the large‐scale climate conditions and state of the physical environment on the Newfoundland and Labrador shelf, and the NWA in general (Cyr & Galbraith, 2021). The NLCI incorporates data from 10 metrics or subindices, including total accumulated ice cover in Atlantic Canada over the entire year (Cyr & Galbraith, 2021) and other physical/environmental traits that might impact, directly or indirectly, food resources available to harp seals. Unfortunately, the linkages and time lags between the physical environmental variables and prey resources are only partially understood. For example, Buren et al. (2014) hypothesized that the mechanistic linkage between sea ice and the modulation of capelin is a match/mismatch phenomenon between the timing of the onset of the spring bloom, which is triggered by ice retreat, and the emergence of *Calanus finmarchicus* (the main prey of capelin) from diapause, with its effects percolating to capelin via nutritional stress. Thus, ice breakup impacts not only capelin recruitment but also the foraging success of YOY learning to forage and even adults building up energy reserves for the next year's reproduction (Stenson et al., 2016). Ideally, an index that more directly describes variation in prey resources would provide clearer insights into harp seal condition, productivity, and YOY survival, but at present, such an index is not available. Further work is therefore needed to better quantify prey availability and the contribution of other environmental factors to juvenile survival.

In addition to climate‐related changes, another major driver of trends since 2000 has been mortality associated with poor ice conditions (Figures 9c and 10a). The ice anomaly index we developed for our model combined information on ice cover when the pups were born (and require stable ice during nursing) with information on ice cover approximately 4 weeks later, when weaned animals require a stable platform for resting during the postweaning fast (Burns et al., 2007; Sergeant, 1991; Stenson & Hammill, 2014). Although separate indices were calculated for each of the Gulf and Front breeding areas, the patterns in ice cover over time were generally similar (Pearson correlation coefficient = 0.45). Information on relative ice cover during the pupping and post‐weaning periods provides a crude measure of platform stability, and combining the two provides insights into ice longevity, which is affected by storm activity and approaching spring breakup. During years with extensive stable ice, the damage to breeding patches from storm activity is dampened, particularly when there is sufficient ice to provide a buffer around areas where pupping occurs (Bajzak et al., 2011; Stenson & Hammill, 2014). In contrast, in years with thinner or less ice cover, wind‐ and storm‐generated swells precipitate ice destruction and breakup, frequently forcing YOY animals into the water, resulting in high mortality (Stenson & Hammill, 2014). The frequency of years with strongly negative ice cover anomalies has increased in recent decades (Figure 2a), and this trend is predicted to continue (Han et al., 2015, 2019).

Although the contribution of human removals (harvest and bycatch) to population trends was highest prior to 1983 (Figure 9a), these hazards continue to be an important source of mortality for harp seals. After more than a decade of very limited harvest numbers, a large‐scale Canadian commercial harvest resumed in 1996 and continued through 2008 at levels not seen since the 1960s (Figure 9a). The increased commercial harvest for YOY, combined with the elevated YOY mortality from environmental factors and ice anomalies, was sufficient to drive a steep population decline beginning in the late 1990s (Figure 5). However, while human removals of adult seals did not increase after 1996 to the same degree as for YOY, adult harvests (primarily in Greenland) still played a significant role in limiting abundance and driving trends (Figure 9c). For long‐lived mammals such as harp seals, population growth is typically far more sensitive to adult mortality than to juvenile mortality (Gerber & Heppell, 2004), and this pattern was confirmed by our LSA results (Appendix S1: Figure S5). Since 1985, the combined hazards associated with human removals contributed more to adult mortality than natural hazards by a factor of more than 5 (Figure 10b), and the largest portion of this mortality was attributable to the Greenland harvest. The effects of this consistent level of harvest mortality for adults contributed less to variation in λ than YOY mortality (Figure 9b,c); yet, harvest removals of adults may be a major factor limiting the potential for recovery during periods when overall YOY mortality decreases (such as after 2015). This highlights the need to carefully manage adult harvests as well as YOY harvests, even though the absolute number of removals may be less.

The effective management and conservation of marine mammal species, particularly those that experience commercial or subsistence harvests, require reliable information about how different factors affect population dynamics. Acquiring such information can be challenging when the data available to monitor populations are incomplete or consist of piecemeal observations of different parameters. A further challenge in a time of rapidly changing environmental conditions is that the factors limiting abundance are unlikely to be consistent over time and will have impacts that may not be detected for several years as a result of lags (e.g., high YOY mortality in 1 year may not be detected until age of recruitment). IPMs, which make use of disparate datasets to elucidate demographic trends and their relationship to environmental or anthropogenic factors (Abadi et al., 2010), provide one solution to this challenge (Schaub & Abadi, 2011; Zipkin & Saunders, 2018). The IPM we present here extends this approach by incorporating competing hazard methods to model survival, allowing us to explore temporal shifts in the magnitude of specific hazards and their relative effects on harp seal vital rates and population trends. Our findings provide novel insights into the dynamics and key determinants of harp seal abundance over the past 70 years. We show how changing levels of human removals of different age classes, combined with a major regime shift and the progressive deterioration of ice cover and environmental conditions in the north Atlantic, have interacted to drive trends in survival and fecundity, and to strongly limit population abundance. Our findings will have important implications for future management decisions. If the population remains low or further declines, we might expect density‐dependent mortality to alleviate. However, most climate models forecast environmental conditions, such as warmer water and decreasing ice cover, which will probably lead to high and possibly increasing levels of mortality. These factors are likely to become the main drivers in the dynamics of this harp seal population unless it can adapt by finding more favorable conditions, possibly further to the north. Changes in environmental and ice conditions are also likely to have impacts on sustainable harvest levels in both Canada and Greenland. Our model can provide a useful tool for exploring future scenarios of climate impacts and management strategies, helping to ensure that this iconic species continues to play an important functional role in North Atlantic food webs.

## AUTHOR CONTRIBUTIONS

Mike O. Hammill and Garry B. Stenson conceived the study. M. Tim Tinker developed and coded the model. Garry B. Stenson compiled and curated the age structure, pregnancy rate, and harvest data. M. Tim Tinker, Mike O. Hammill, and Garry B. Stenson wrote the first draft. All authors participated in additional analyses, provided critical insights into the drafts and approved the manuscript for submission.

## CONFLICT OF INTEREST STATEMENT

The authors declare no conflicts of interest.

## Supporting information


Appendix S1.

## Data Availability

Data and code (Tinker et al., 2025) are available in Dryad at https://doi.org/10.5061/dryad.2z34tmq0k.
