# Supplementary material for: Drivers of population dynamics and juvenile mortality in Northwest Atlantic harp seals
Source: Ecol Appl. 2026 Feb 20;36(1):e70184. doi: 10.1002/eap.70184 (PMC12922475; doi:10.1002/eap.70184)
Supplement: Supplementary file 1 — Appendix S1. [file EAP-36-e70184-s001.pdf]

Title: Drivers of population dynamics and juvenile mortality in Northwest Atlantic harp seals

Authors: M. Tim Tinker, Garry B. Stenson, Arnaud Mosnier, Joanie Van de Walle, Shelley L.C. Lang and Mike O. Hammill

Journal: Ecological Applications

Appendix S1: Supporting Information (SI) methods, figures and tables

Section S1: SI Methods

Scaling constants for calculating hazard rates

To facilitate the fitting of parameters that determine hazard rates (α, β, ϕ, γ), we included scaling

constants in equations 8, 9, 11, 14, 15 and 16 of the manuscript. In the interest of clarity these constants were not shown in the main manuscript equations, but are represented by the symbol "ϖ" in the full versions of these equations presented here:

$$h_0(t) = \exp\left(\varpi + \gamma_0 + \gamma_{D0}\right) \tag{8*}$$

$$h_A(i,t) = \exp\left(\varpi + \gamma_A(i) + \gamma_{DA}\right) \tag{9*}$$

$$h_{IC}(t) = \sum_{a=1}^{3} P(a,t) \cdot \exp\left(\varpi + (1-\varpi) \cdot \gamma_{IC}(a,t)\right) \tag{11*}$$

$$h_{H0,j}(t) = \exp\left(\varpi + \gamma_{H0,j}(t)\right) \tag{14*}$$

$$h_{HA,j}(t) = \exp\left(\varpi + \gamma_{HA,j}(t)\right) \tag{15*}$$

$$F(i,t) = \begin{cases} \exp\left[-\exp\left(\varpi + \beta_1 + \beta_2 \cdot (8-i)^2 + \phi_F \cdot N(t-1) \cdot \exp\left\{\delta \cdot NLCI(t-1) + \varepsilon_F(t)\right\}\right)\right] & \text{if } 4 \le i \le 8 \\ \exp\left[-\exp\left(\varpi + \beta_1 + \phi_F \cdot N(t-1) \cdot \exp\left\{\delta \cdot NLCI(t-1) + \varepsilon_F(t)\right\}\right)\right] & \text{if } i > 8 \end{cases} \tag{16*}$$

We set ϖ constants *a priori* to values corresponding to improbably small hazards for the vital rate in question: for equation 16* (which determines fecundity) we set $\varpi = -3$, while for the remaining equations (which determine survival) we set $\varpi = -10$. The inclusion of scaling factors facilitated model fitting and interpretation by allowing us to set non-informative, half-Cauchy priors on log-hazard parameters α, β, ϕ, γ (Table S6), estimates for which now represent log hazard ratios relative to ϖ. In the absence of useful empirical information to inform the value of a given log hazard parameter, the half-Cauchy prior for that parameter will shrink towards 0, thus defaulting to the negligibly small hazard rate implied by ϖ. Although values of ϖ were chosen somewhat arbitrarily, they were set low enough that the estimation of log-hazard parameters was effectively unconstrained and thus hazard rate estimates were fully data-driven and not influenced by the choice of ϖ value (i.e., shifting the value of ϖ up or down had absolutely no effect on the estimated hazard/survival rates).

Prior selection for specific parameters

Most parameters of the process model were set with vague Cauchy or half-Cauchy parameters (Table S6); however, we set moderately informative priors for specific parameters (ψ, ζ, π, and υ) based on published information and/or biological feasibility, and to improve model identifiability. For ice-anomaly effect parameters we used normal priors with mean = 3 and SD = 1.5 (for ψ_0) and mean = 1.5 and SD = 1 (for ψ_a), as these values produced a functional form consistent with previous reports of ice anomaly effects on YOY survival (Stenson & Hammill 2014) yet allowed for flexibility in the magnitude of impacts (e.g., for a substantial negative anomaly of -2.5, the inter-quartile range for prior expectation of mortality was 0.001 – 0.75). For parameter ζ, which scales the magnitude of density-dependent and environmental impacts on survival for older age classes relative to YOY, we used a beta prior with mean = 0.1 and SD =

0.04, assuming density-dependent and environmental impacts on adults were much lower than for YOY, as is typical for long-lived mammals (Eberhardt 2002). Parameter $\pi(t)$ was drawn from a beta prior distribution having mean = 0.05 and SD=0.03, based on previous work suggesting that newborn mortality is generally quite low (Sergeant 1991). For parameter υ, which scales YOY natural hazards relative to those of older animals, we used a normal prior with mean = 1.1 and SD = 0.2, corresponding to previously reported estimates of the ratio of pup mortality to adult mortality at low population density (mean = 3x, 99% quantiles = 1.8 - 5x; Roff and Bowen 1983, 1986). A table of prior distributions for all parameters is provided in Table S6.

Life stage simulation analysis (LSA)

We used Bayesian life stage simulation analysis (LSA) to assess the effects of different hazards on harp seal population dynamics. Our methods follow those described originally by Wisdom et al. (2000) and elaborated for a Bayesian population model by Eaker et al. (2017). We had two objectives in conducting this LSA: 1) to assess the sensitivity of population growth rate (λ) to proportional perturbations in cause-specific hazard rates and fecundity (we back-transformed fecundity to instantaneous hazard units for comparison to survival hazards); and 2) to evaluate the degree to which actual variation in each hazard rate contributed to observed variation in λ . We repeated the analyses for each of four time periods (*p*): i) 1951-1982, ii) 1983-1999, iii) 2000-2020, and iv) 1951-2020 (i.e. the entire study period). The first 3 focal periods were selected to represent the early, middle and late portions of the overall study period, with breakpoints corresponding approximately to management-based shifts in harvest activity.

For each of the four focal periods defined above, we iterated the following steps 1000 times:

1) We drew random values for all base parameters from the joint posterior distribution of the fitted model.
2) We combined the randomly drawn parameter values with the environmental variables (NLCI and IC indices) to compute values for each cause-specific hazard rate and fecundity for each year of the focal period, by solving equations 2 – 16 as described in the main manuscript.
3) We fit kernel density distributions to each set of hazard rate values across time within the focal period. These distributions describe temporal variation in each hazard rate: we used non-parametric kernel density distributions because in some cases the distribution of values was not well fit by parametric distributions.

4) We then generated 1000 “random but plausible” sets of vital rates for the focal period, by solving equations 2, 3 and 16 using hazard rate values drawn from the kernel density distributions calculated in step 3. Each of the resulting sets of random vital rates corresponds to a potential set of environmental and demographic conditions based on the input variables and parameter estimates for that focal period.

5) For each set of random vital rates, we assembled a population matrix (equation 17) and used algebraic methods to estimate the associated asymptotic annual growth rate (λ), calculated as the dominant eigenvalue of the matrix (Caswell 2001).

6) Using results from steps 4 and 5, we fit a generalized linear model (GLM) to estimate the functional relationship between λ and 12 hazard rates: fecundity (back-transformed to a hazard rate), natural YOY hazards, natural adult hazards, ice-anomaly hazards, and each of the four human removal hazards (arctic harvest, bycatch, Canadian commercial harvest, Greenland harvest) for YOY and for adults.

7) We calculated two sets of statistics from the fitted GLM: i) the slope coefficients for each hazard rate, which describe the proportional change in λ associated with a corresponding proportional change in each hazard rate (assuming all other hazard rates were held at their mean values); and ii) the partial R^2 values, which describe the proportion of variance in λ explained by variation in each hazard rate, after accounting for the effects of all other hazards. The first statistic represents the scaled sensitivities of λ to each hazard rate (i.e., similar to elasticities in a conventional matrix sensitivity analysis), while the second statistic represents a retrospective analysis of how much each hazard rate contributed to variation in λ during focal period p.

The results of steps 1-7 were averaged across the 1000 draws from the joint posterior, in order to account for parameter uncertainty. We then created box plots to compare the distributions of coefficients and partial R^2 values between different hazard rates. Because the coefficients for hazard rates did not vary noticeably across study periods, we only show values for $p = 4$ (all years of the study period), and we plot the average coefficient values for YOY removals and adult removals because the source-specific coefficient values were almost identical (Figure S5).

We note that asymptotic lambda estimates generated by the Bayesian LSA (λ_{sim}) are distinct from realized population growth rates (λ_{obs}) in the historical, time-variant environment, as the latter also reflect transient dynamics and lag-related effects of shifting age structure. To ensure that the distributions of simulated growth rates for each period were indeed reflective of the time-variant, realized growth rates (calculated as $\lambda_{obs,t} = N(t+1) / N(t)$), we graphically compared the distributions of λ_{obs} and λ_{sim} for each focal period (Figure S6) and tested for differences using t-tests with α=.05 and a Benjamini-Hochberg adjustment for multiple comparisons (λ values were log-transformed for normality and unequal variances accommodated using Welch's t-test).

Data on human removals (harvest and bycatch)

Northwest Atlantic harp seals are taken by commercial and subsistence hunters in the Atlantic Canada waters off southern Labrador and/or the Northeast coast of Newfoundland (‘the Front’- NAFO Divisions 2J and 3KL), in the Gulf of St. Lawrence (‘the Gulf’-NAFO Division 4RST), off western and southeastern Greenland (NAFO Division 1A-F; ICES Area XIVb), and in the eastern Canadian Arctic (primarily along the east coast of Baffin Island).

For each source of human removal (see Table S4), there are recognized uncertainties in the reported numbers of removals by year and, likewise, in the proportion of the reported removals composed of young of the year (YOY) versus seals 1 year of age and older (referred to as adults). In most cases the magnitude of these uncertainties has varied over the course of the time series. In previous population modelling of harp seals (Tinker et al. 2023), a single coefficient of variation (CV) of 0.1 was used to account for uncertainties in reporting for all sources of removals over all years. For the current model, we quantified the degree of uncertainty in total harvest numbers and age structure (proportion YOY) on an annual basis for each mortality source, based on previously published reports (Stenson 2010, Stenson and Upward 2020) and expert opinion.

For total removals and the proportion of YOY, we classified the level of certainty for each source of removals as “high”, “moderate” or “low”. For total removals, we assumed that a high level of certainty corresponds to a CV of 0.05, a moderate level of certainty corresponds to a CV of 0.1 (i.e., the default value used for previous assessments), and a low level of certainty corresponds to a CV of 0.2. For the proportion of YOY, uncertainty around the reported proportion of the harvest consisting of YOY (PYOY) is described using a beta distribution with parameters $a = PYOY \times \kappa$ and $b = (1 - PYOY) \times \kappa$, where κ is a precision parameter ($\kappa > 0$,

higher values of κ = reduced variance). As with total harvest numbers, we assigned one of three levels of certainty (high", "moderate" or "low") to the PYOY values for each year and for each source or removals. Values of κ were set to 25, 100 or 2500 for low, medium or high certainty (respectively), corresponding to standard deviations of 0.1, 0.05 and 0.01 for PYOY = 0.5. These κ values were selected to correspond to the 95th quantiles of feasible values for reported PYOY values. In the sections below we describe the sources of data for total removals and the proportion of YOY in each source of removal. This information, based on multiple sources (described below) and the expert opinion of coauthors G. B. Stenson and M. O. Hammill, served as the basis for the attribution of uncertainty levels for each source of removal (Table 5, and see main paper).

Arctic Canada

Total removals

Annual removals for the period 1952-1982 were taken from Bowen (1982) and Roff and Bowen (1986). Removals for the period 1983-1996 were unknown and the estimated catch in 1982 was used for annual removals for this period. For the period 1997-2001, data from a five year study of marine mammal harvest in Nunavut based upon interviews in each community (Anon. 2005) was used. No information is available for the period 2002-2019. Hence, the average catch estimated during the five-year study (715 per year) was rounded to 1,000 and assumed to apply to this entire period.

Proportions of YOY

Roff and Bowen (1986) reported that approximately 3% of the harvest was comprised of YOY. As there is no information about how (and if) this proportion has changed over time, the 3% YOY in the harvest was assumed for 1952-2019.

Level of uncertainty

There is very limited data on catch numbers and the proportion of YOY in the catches in the Canadian Arctic harvest (Stenson 2010). Because of this, we attributed a "high" level of uncertainty around catch numbers and proportion of YOY in the catches for 1952-2019.

Greenland Harvest

Total removals

Catches for the period 1952-1953 were estimated by Bowen (1982). Reported catches were used for 1954-2017 from ICES (2019), except between 1988-1992 when no data were available. For this period, catches were estimated by linear interpolation between the available data following Stenson (2014). Data from 1993-2019 were updated from Stenson and Upward (2020) and ICES (2023) based on recent data obtained from the Greenland Institute of Natural Resources.

Proportions of YOY

For the periods 1952-1962 and 1963-1969, data was taken from Bowen (1982). For the period 1970-1983, with the exception of 1981, data was taken from Kapel (1999). For year 1981, data from 1980 was used (Stenson et al. 1999). For 1984-1993, although samples were collected, sample sizes were small and not necessarily collected for the purpose of age composition evaluation. For 1984-2019, an average from combined data collected in central and northwest Greenland over 1984-1991 and in southwest Greenland between 1986-1993 was used instead to estimate age composition. Greenland reports the proportion of young (1 to approximately 5 year-old) and older animals in the harvest. The proportion of young varies between 10-40% between years. As a result, the proportion of YOY cannot be higher that 40%.

Level of uncertainty

Removals in 1952-1953 were estimated and as such were considered of "moderate" uncertainty. There is little uncertainty in the total removals for 1954-1988 as reported catches were used. For 1998-1992, when no data were available, a high level of uncertainty was given. In 1993 the methods of collecting catch data changed from being centrally reported by an individual in each community to self reporting by individual hunters. We assumed this latter method would have greater uncertainty than catches from 1954-1988 and a "moderate" level of uncertainty was given for that period.

Because the proportion of YOY in the catches for the period 1952-1969 was based on some (albeit limited) data, this period was given a "moderate" uncertainty level. For the period 1970-1983, the level of uncertainty in the proportion of YOY was low because it was based on observed data obtained from larger sample sizes and over a wider distribution. We considered that the level of uncertainty was high from 1984 onward in the proportion of YOY in the sample because it represents an average of values observed during the period 1970-1983 and it is possible that this proportion has changed over time.

<u>Canadian Commercial Harvest (Atlantic Canada)</u>

Total removals

Total catches at the Front and in the Gulf for the years 1952-78 were compiled from values reported in the Statistical Bulletin of the International Commission for Northwest Atlantic Fisheries (ICNAF 1970-1977). Total catches for the years 1979-89 were compiled from values reported in the Statistical Bulletin of the Northwest Atlantic Fisheries Organization (NAFO 1984-94). Total catches at the Front and in the Gulf for the years 1990-2019 were provided by the DFO Statistics Branch.

Proportions of YOY

The catch statistics provided by ICNAF, NAFO and the DFO Statistical Branch are reported according to pelage type. Based upon these reports, Front and Gulf catches can be split into YOY and adults (age 1+). The age structures of catches during the 1952-83 period were taken from Bowen (1982) and Roff and Bowen (1986). For the period 1984-2019, the proportion of YOY seals taken in the harvest was taken from data provided by the Statistics Branch. This is the same approach that has been taken for all previous analyses (Bowen 1982, Roff and Bowen 1986, Sjare et al. 1996, Stenson et al. 1999, 2000, Stenson 2005, Stenson 2010). The only exceptions occurred in 1998 and 1999 when a portion of the catch was not identified according to pelage. The age of 7 % of the catch was not identified in 1998. It was assumed that the proportion of YOY in this catch was the same as for the remainder of the catch for which ages were available. In 1999, approximately 22 % of the catch did not have assigned ages. As these animals were all from the Gulf of St. Lawrence, the age structure of seals taken by the small boats in the Gulf (which were reported by age) was used.

Level of uncertainty

From 1952-1970, data on total removals was considered with moderate uncertainty since monitoring of the hunt was not extensive. From 1971-2019, which is a period when quotas were implemented, data on total removals were considered with low uncertainty because catches were better monitored during this period. For the proportion of YOY in the catches, the level of uncertainty was considered moderate for the period 1952-1964. In 1965 restrictions on hunting females were implemented and as a result of increased monitoring, we consider the uncertainty on the reported data to be low for the period 1965-2019.

Bycatch

Total removals

Harp seals are taken as incidental bycatch in the spring Newfoundland lumpfish fishery and in the U.S. fisheries. Those sources of data are combined to yield a summed annual bycatch. There is no information on bycatch prior to 1970.

For the lumpfish fisheries, bycatch numbers for the period 1970-2003 were estimated by Sjare et al. (2005). A study using data from 1989 to 2003 estimated the rate of bycatch (number of seals bycaught per ton of roe)(Sjare et al. 2005). Bycatch from the beginning of the lumpfish fishery in 1970 until 1988 was estimated based upon mean bycatch levels from 1989-1991 (i.e., the initial period of the study). The average bycatch rate for the last five years of the study (1999-2003) were used to estimate bycatch numbers for 2004-2018 by Stenson and Upward (2020) and also for 2019 based on lumpfish landings obtained from DFO Statistics Branch.

Data on incidental catches of harp seals in U.S. fisheries for the period 1970-2005 were summarized by Waring et al. (2005, 2007). For the period 2006-2011, data was taken from Waring et al. (2013) and Waring et al. (2014). For the period 2012-2016, data was taken from Hayes et al. (2019). For the period 2017-2019, data was taken from the most recent assessments in the US for harp seals (Hayes et al. 2022).

Proportions of YOY

Sjare et al. (2005) estimated the proportion of YOY seals bycaught from 1989-2000 using age class records provided by fishers over that time period. As in Sjare et al. (2005), the average age classes from 1989 to 1991 were applied to the 1970-1988 period while averages for 1996 to 2000 were applied to 2000-2019 as in Stenson and Upward (2020).

Level of uncertainty

Since there is no bycatch data prior to 1970, but bycatch could still have happened during the period 1952-1970 in other fisheries, we considered our uncertainty towards that source of information as “moderate” for that period. Total removals from bycatch are, for the most part, extrapolations based on fishery yields and were thus considered with “moderate” uncertainty throughout, except in 1989-2003, where actual data were collected. The proportion of YOY in the bycatch is assumed to be known with low uncertainty throughout.

References

Anonymous. 2005. The Nunavut Wildlife Harvest Study. Nunavut Wildlife Management Board. Iqaluit Nunavut.

Bowen, W.D. 1982. Age structure of Northwest Atlantic Harp Seal catches, 1952-80. NAFO Sci. Coun. Studies 3: 53-65.

Bowen, W.D., Capstick, C.K. and Sergeant, D.E., 1981. Temporal changes in the reproductive potential of female harp seals (Pagophilus groenlandicus). Canadian Journal of Fisheries and Aquatic Sciences, 38(5), pp.495-503.

Caswell, H. 2001. Matrix population models: construction, analysis, and interpretation. 2nd ed. Sinauer Associates, Sunderland, MA.

Eacker, D. R., P. M. Lukacs, K. M. Proffitt, and M. Hebblewhite. 2017. Assessing the importance of demographic parameters for population dynamics using Bayesian integrated population modeling. Ecological Applications 27:1280–1293.

Eberhardt, L.L. 2002. A paradigm for population analysis of long-lived vertebrates. Ecology 83:2841-2854.

Fisher, H.D. 1954. Studies on reproduction in the harp seals (*Phoca groenlandica* Erxleben) in the Northwest Atlantic. Ph.D. Thesis Dept., of Zoology, McGill University, Montreal, QC, Canada.

Frie, A.K., K.-A. Fagerheim, M.O. Hammill, F.O. Kapel, C. Lockyer, C., et al. 2011. Error patterns in age estimation of harp seals (Pagophilus groenlandicus): results from a transatlantic, image-based blind-reading experiment using known-age teeth. ICES Journal of Marine Science 68:1942-1953.

Hammill, M.O., G.B. Stenson, A. Mosnier and T. Doniol-Valcroze. 2021. Trends in abundance of harp seals, Pagophilus groenlandicus, in the Northwest Atlantic, 1952-2019. DFO *Canadian Science Advisory Secretariat Research Document* 2021/006. iv + 30 p.

Hayes, S.A., Josephson, E., Maze-Foley, K., and P.E. Rosel (eds.). 2019. US Atlantic and Gulf of Mexico Marine Mammal Stock Assessments – 2018. NOAA Technical Memorandum NMFS-NE-258.

Hayes, S.A., Josephson, E., Maze-Foley, K., P.E. Rosel, and J. Wallace (eds.). 2022. US Atlantic and Gulf of Mexico Marine Mammal Stock Assessments – 2021. NOAA Technical Memorandum NMFS-NE-288.

Kapel, F.O. 1999. Age composition in Greenland catches of harp seal. Working paper presented to the national Marine mammal Peer Review Committee, Montreal, Canada, Feb. 1-5, 1999.

ICES. 2019. ICES/NAFO/NAMMCO Working Group on Harp and Hooded Seals (WGHARP).

ICES. 2023. Joint ICES/NAFO/NAMMCO Working Group on Harp and Hooded Seals (WGHARP). ICES Scientific Reports. https://doi.org/10.17895/ices.pub.24306100.v1

CES Scientific Reports. 1:72. 193 pp. *http://doi.org/10.17895/ices.pub.5617*.

ICNAF. 1970. Sealing statistics for 1937-68. ICNAF Stat. Bull. 18: 124-140.

1971. Sealing statistics for 1969. ICNAF Stat. Bull. 19: 119-120.

1972a. Sealing statistics for 1970. ICNAF Stat. Bull. 20: 109-110.

1972b. Sealing statistics for 1971. ICNAF Stat. Bull. 21: 133-134.

1974. Sealing statistics for 1972. ICNAF Stat. Bull. 22: 215-216.

1975. Sealing statistics for 1973. ICNAF Stat. Bull. 23: 241-242.

1976. Sealing statistics for 1974. ICNAF Stat. Bull. 24: 253-254.

1977. Sealing statistics for 1975. ICNAF Stat. Bull. 25: 229-230.

NAFO. 1984a. Sealing statistics for 1979. NAFO Stat. Bull. 29: 291-292. (Revised)

1984b. Sealing statistics for 1980. NAFO Stat. Bull. 30: 277-278. (Revised)

1984c. Sealing statistics for 1982. NAFO Stat. Bull. 32: 283-284.

1985a. Sealing statistics for 1981. NAFO Stat. Bull. 31: 275-276. (Revised)

1985b. Sealing statistics for 1983. NAFO Stat. Bull. 33: 277-278.

1986. Sealing statistics for 1984. NAFO Stat. Bull. 34: 303-304.

1987. Sealing statistics for 1985. NAFO Stat. Bull. 35: 319-320.

1989. Sealing statistics for 1986. NAFO Stat. Bull. 36: 303-304.

1990. Sealing statistics for 1987. NAFO Stat. Bull. 37: 293-294.

1991. Sealing statistics for 1988. NAFO Stat. Bull. 38: 303-304.

1993. Sealing statistics for 1989. NAFO Stat. Bull. 39: 297-298.

1994. Sealing Statistics for 1990. NAFO Stat. Bull. 40: 307-308.

Roff, D.A., and Bowen, W.D. 1983. Population dynamics and management of the Northwest Atlantic harp seal (Phoca groenlandica). Can. J. Fish. Aquat. Sci. 40: 919-932.

Roff, D.A. and W.D. Bowen. 1986. Further analysis sf population trends in the northwest Atlantic harp seal (Phoca groenlandica) from 1967 to 1985. *Canadian Journal of Fisheries and Aquatic Science* 43: 553-564.

Sergeant DE 1991. Harp seals man and ice. *Canadian Special Publication of Fisheries and Aquatic Sciences.* 114: 153 p

Sergeant, D.E. and Fisher, H.D. 1960. Harp seal populations in the western North Atlantic from 1950 to 1960. Fisheries Research Board of Canada, Arctic Unit.

Sjare, B., Stenson, G.B., and Wakeham, D. 1996. Summary of the catch and catch-at-age data for harp seals in the Northwest Atlantic, 1946-94. NAFO Sci. Coun. Studies 26: 33-39.

Sjare, B., Walsh, D., Benjamins, S., and Stenson, G.B. 2005. An update of estimated harp seal by-catch in the Newfoundland lumpfish fishery. DFO Can. Sci. Advis. Sec. Res. Doc. 2005/049.

Smith, T.G., and Taylor, T. 1977. Notes on marine mammal, fox and polar bear harvests in the Northwest Territories 1940 to 1972. Fish. Mar. Serv. Tech. Rep. No. 694.

Stenson, G.B. 2005. Estimates of human induced mortality in Northwest Atlantic Harp Seals, 1952-2004. DFO Can. Sci. Advis. Sec. Res. Doc. 2005/050.

Stenson, G. B. 2010. Total removals of Northwest Atlantic harp seals (Pagophilus groenlandicus) 1952-2009. Canadian Science Advisory Secretariat Research Document, 2009/112, iv+30p.

Stenson, G.B. 2014. Updated estimates of Harp Seal removals in the Northwest Atlantic. DFO Can. Sci. Advis. Sec. Res. Doc. 2014/015.

Stenson, G.B., Buren, A.D., and M. Koen-Alonso. 2016. The impact of changing climate and abundance on reproduction in an ice-dependent species, the Northwest Atlantic harp seal, Pagophilus groenlandicus. *ICES Journal of Marine Science* 73:250-262.

Stenson, G., J-F Gosselin, J. Lawson, A. Buren, P. Goulet, et al. 2022. Pup production of harp seals in the Northwest Atlantic in 2017 during a time of ecosystem change. NAMMCO Scientific Publications 12. https://doi.org/10.7557/3.6214

Stenson, G., and M. Hammill. 2014. Can ice breeding seals adapt to habitat loss in a time of climate change? *ICES Journal of Marine Science* 71:1977–1986.

Stenson, G.B., Healey B., Sjare, B., and Wakeham, D. 2000. Catch-at-age of northwest Atlantic harp seals, 1952-1999. DFO Can. Stock. Assess. Sec. Res. Doc. 2000/079.

Stenson, G.B., Sjare, B., and Wakeham, D. 1999. Catch-at-Age of Northwest Atlantic Harp Seals. DFO Can. Stock Assess. Sec. Res. Doc. 99/105.

Stenson, G. B., & Upward, P. 2020. Updates estimates of harp seal bycatch and total removals in the Northwest Atlantic. Canadian Science Advisory Secretariat Research Document (2020/014).

Tinker, M.T., Stenson, G.B., Mosnier, A., and Hammill, M.O. 2023. Estimating Abundance of Northwest Atlantic Harp Seal Using a Bayesian Modelling Approach. DFO Can. Sci. Advis. Sec. Res. Doc. 2023/068. iv + 56 p. ISBN 978-0-660-68920-3

Waring, G.T., Josephson, E., Fairfield, C.P., and K. Maze-Foley. 2006. Draft U.S. Atlantic and Gulf of Mexico marine mammal stock assessments- 2005. NOAA Tech. Mem. NMFS-NE194.

Waring, G.T., Josephson, E., Fairfield, C.P., and K. Maze-Foley. 2007. Draft U.S. Atlantic and Gulf of Mexico marine mammal stock assessments- 2005. NOAA Tech. Mem. NMFS-NE-205.

Waring, G.T., Josephson, E., Maze-Foley, K., and P.E. Rosel (eds.). 2011. U.S. Atlantic and Gulf of Mexico marine mammal stock assessments-2010. NOAA Tech. Mem. NMFS-NE-219.

Waring, G.T., Josephson, E., Maze-Foley, K., and P.E. Rosel (eds.). 2013. U.S. Atlantic and Gulf of Mexico marine mammal stock assessments-2012. NOAA Tech. Mem. NMFS-NE-223.

Waring, G.T., Josephson, E., Maze-Foley, K., and P.E. Rosel (eds.). 2014. U.S. Atlantic and Gulf of Mexico marine mammal stock assessments-2013. NOAA Tech. Mem.

Wisdom, M. J., L. S. Mills, and D. F. Doak. 2000. Life stage simulation analysis: Estimating vital-rate effects on population growth for conservation. Ecology 81:628–641.

Supplementary Figures

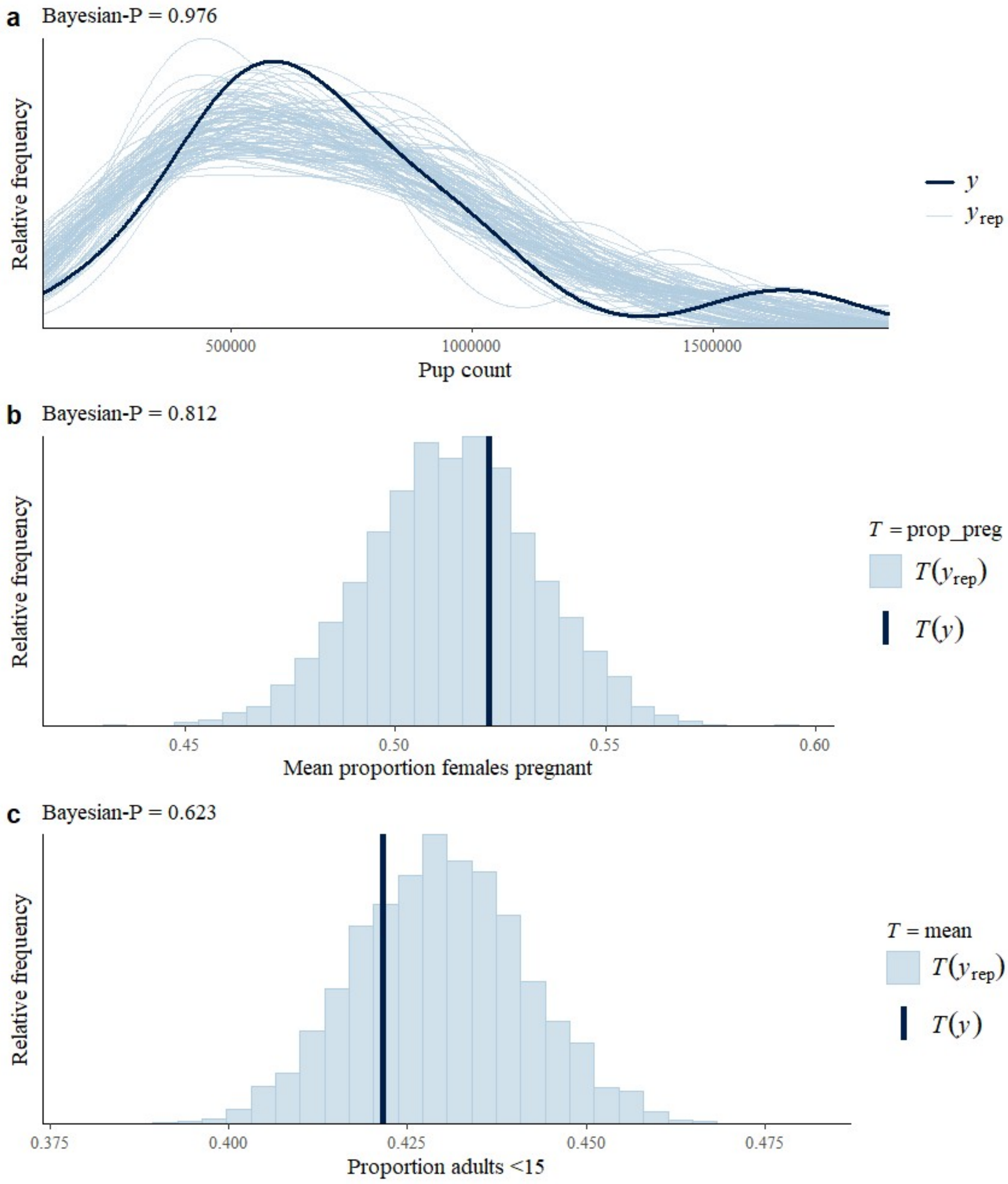

Figure S1. Graphical representation of posterior predictive check (PPC) diagnostics: a) median pup counts based on observed data (black line, “y”) compared to histogram of replicate out-of-sample or “new” observations (light grey bars, “yrep”) generated by the model; b) median proportion of females pregnant (black vertical line, “T(y)”) compared to a histogram of equivalent mean values for replicate out-of-sample or “new” observations (light grey bars, “T(yrep)”); c) median proportion of adults < 15 years of age (black vertical line, “T(y)”) compared to a histogram of equivalent mean values for replicate out-of-sample or “new” observations (light grey bars, “T(yrep)”).

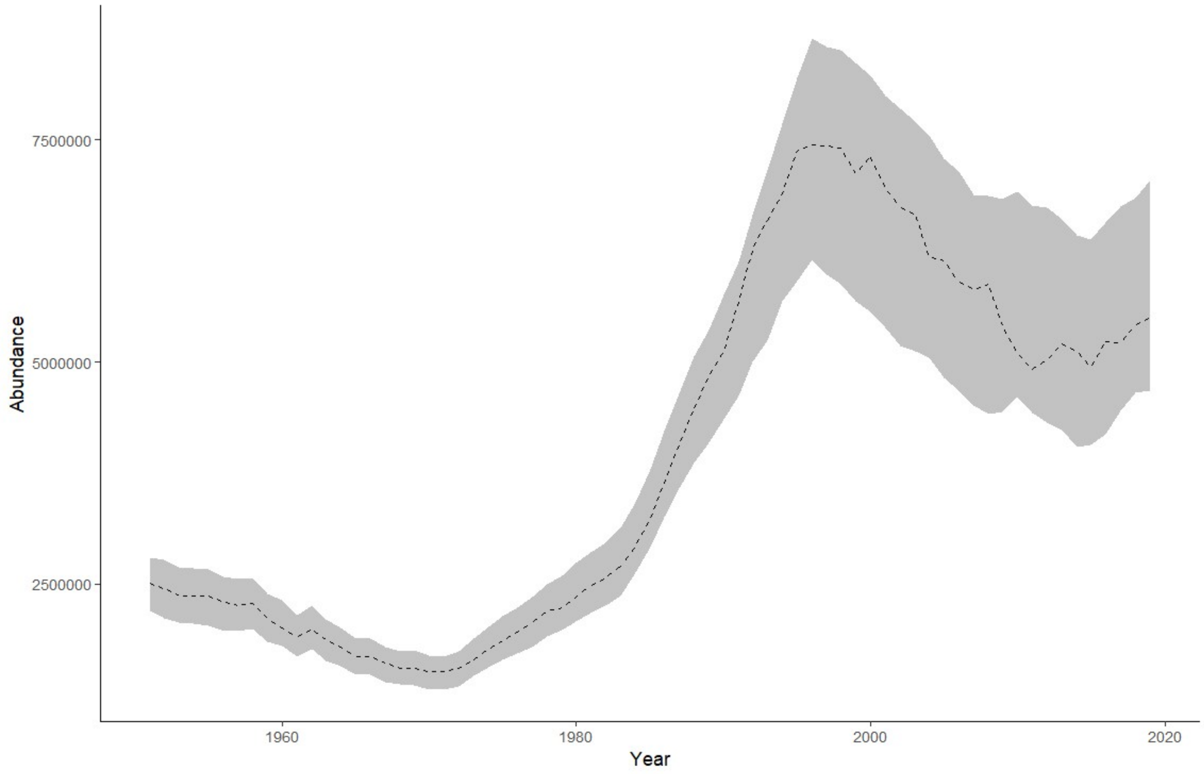

Figure S2. Distribution of hindcast posterior predictive simulations of population abundance (grey band encloses 95% of simulations), with the model-estimated trend shown as a dashed black line.

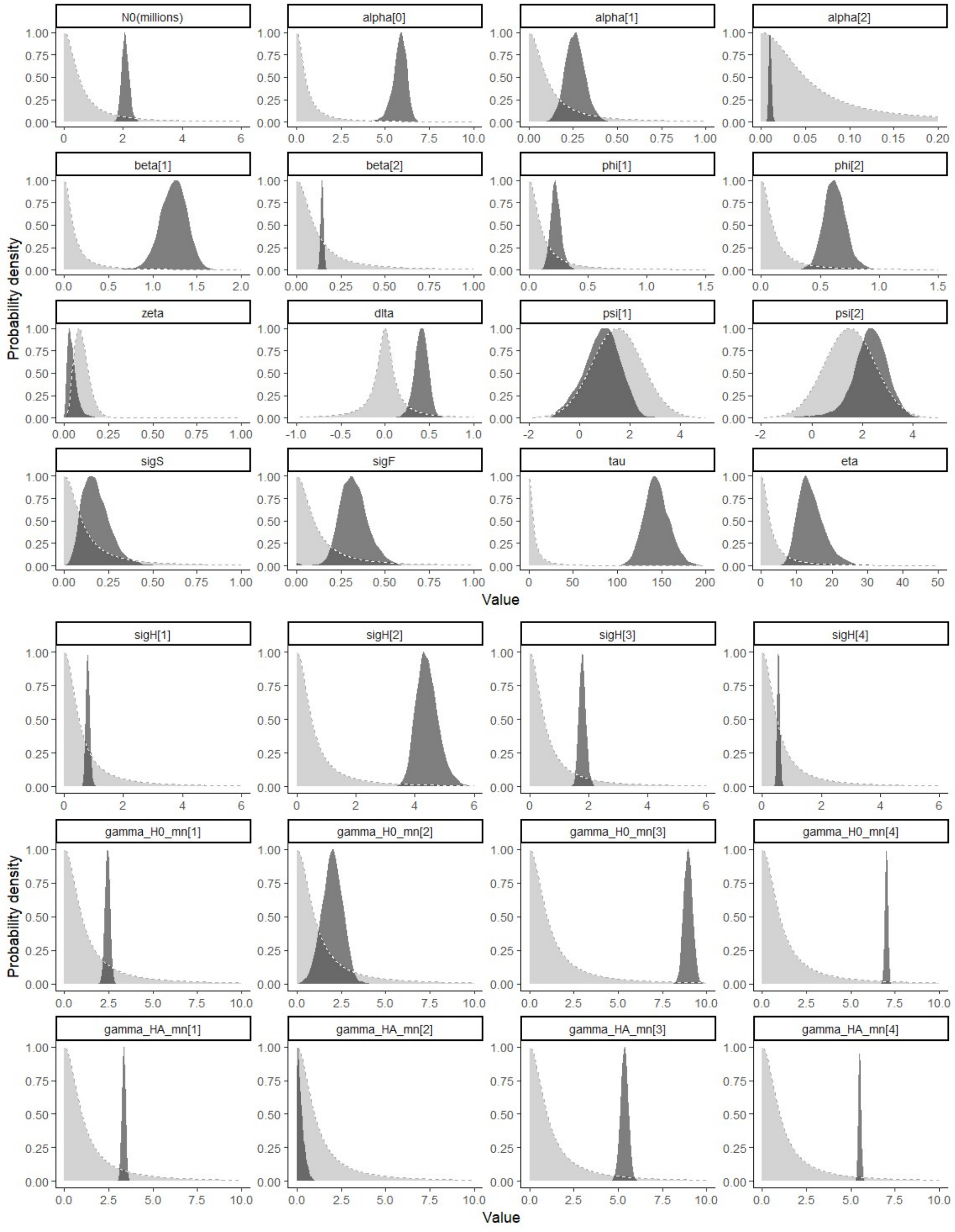

Figure S3. Comparison of prior distributions (light grey shaded areas) to posterior distributions (dark grey shaded areas) for model parameters. Not shown here are distributions for parameters υ and ψ_0, for which the informative priors (Table S6) and posteriors were essentially identical. Also not shown are hierarchical parameters based on the hyper-parameters shown here. Note that half-Cauchy prior distributions are left-bounded by 0.

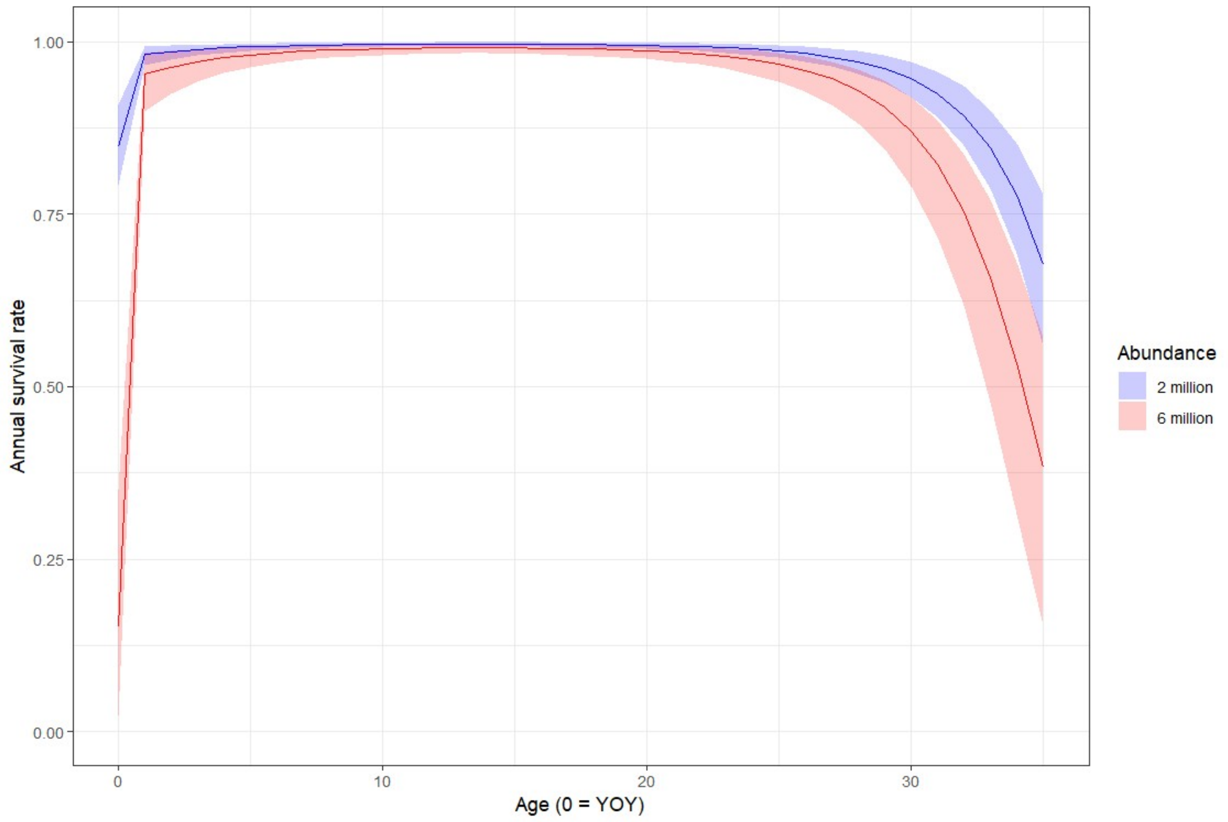

Figure S4. Model-estimated relationship between annual survival rates and age, based on results of the fitted integrated population model. Solid lines indicate mean estimated values and shaded bands indicate the associated 95% CI. The age-specific survival curves are plotted for two population densities (low = 2 million, high = 6 million) to illustrate density-dependent effects.

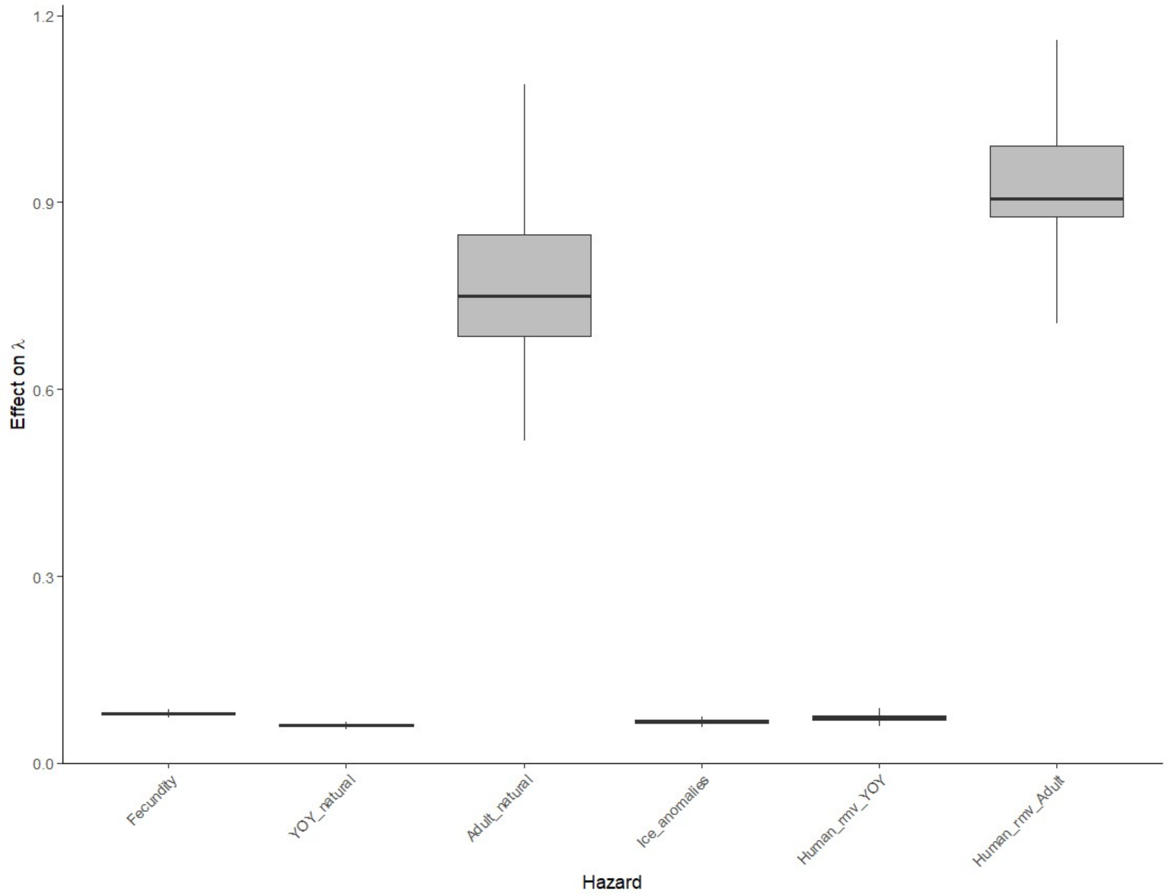

Figure S5. Results from Bayesian Life Stage Analysis (LSA) showing the sensitivity of lambda to proportional perturbations in the instantaneous hazard rates for fecundity and cause-specific mortality for YOY and adults. Sensitivities represent the individual coefficients from a generalized linear model (GLM) of lambda vs. hazard rates, fit to iteratively generated projections of the process model in which base parameters were drawn from the joint posterior and temporal variation in each hazard (reflecting variation in environmental conditions, ice cover, and human removals) were simulated based on distributions of estimated values for each hazard rate across years.

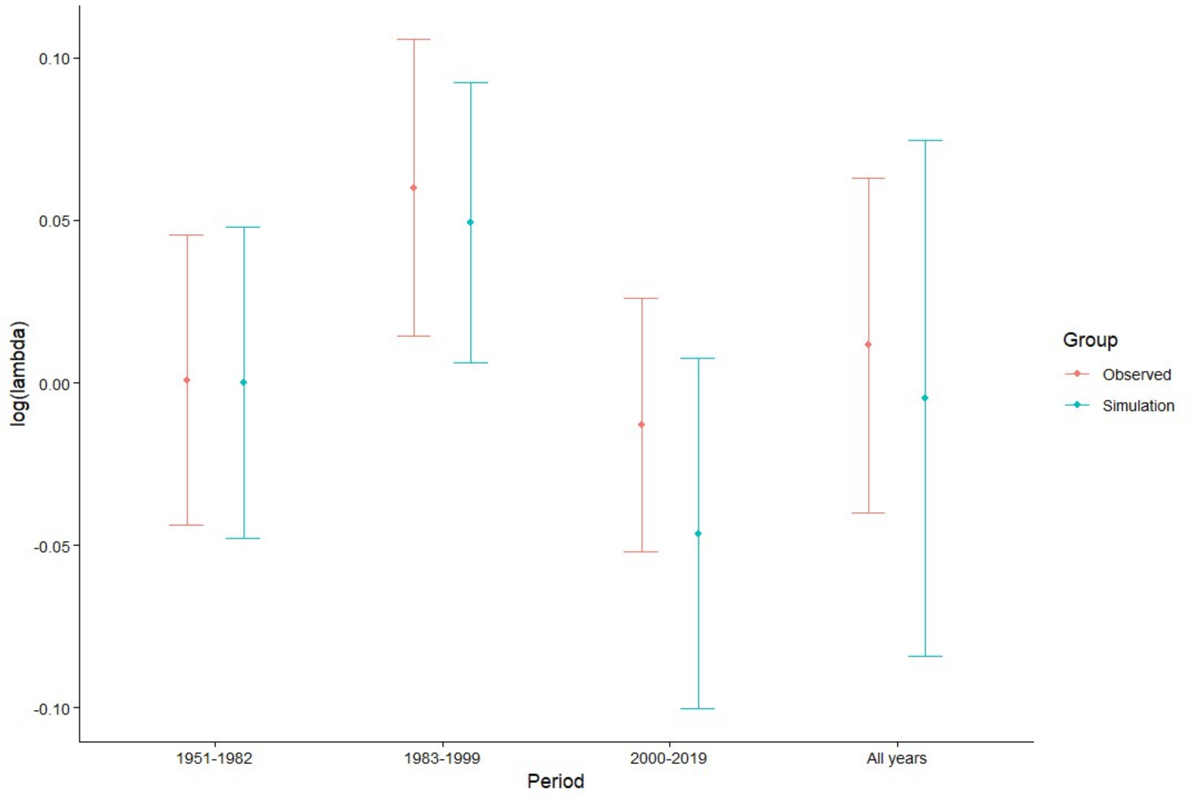

Figure S6. Comparison of distributions of 2 groups of log-transformed population growth rate estimates (λ) computed for 4 focal periods. The first group ("observed" growth rates, λ_{obs}) represents the distribution of model-estimated, realized population growth rates for the historical, time-variant environment (calculated as $\lambda_{obs,t} = N(t+1) / N(t)$). The second group ("simulation" growth rates, λ_{sim}) represents the distribution of asymptotic growth rates generated by the Bayesian LSA simulations. The mean values of λ_{sim} for each period were within 1 standard deviation of the mean λ_{obs} values, and the two distributions did not differ significantly for any of the 4 focal periods (t-test P = 0.938, 0.648, 0.126 and 0.307).

Supplementary Tables

Table S1. Pup (YOY) production estimates and the percentage of pupping occurring in each area (1990 onward) used as input into the population model. A, Aerial surveys, M-R, mark-recapture based estimates.

Year	Type	Total (SE)	Southern Gulf (%)	Northern Gulf (%)	Front (%)	Reference
1951	A	645,000 (322,500)[1]	-	-	-	Sergeant and Fisher 1960
1960	A	235,000 (117,500)[1]	-	-	-	
1978	M-R	497,000 (68,000)	-	-	-	Roff and Bowen 1986
1979	M-R	478,000 (70,000)	-	-	-	
1980	M-R	475,000 (94,000)	-	-	-	
1983	M-R	534,000 (66,000)	-	-	-	
1990	A	577,900 (38,800)	18	1	81	Stenson et al 2022
1994	A	702,900 (63,600)	28	8	64	
1999	A	997,900 (102,100)	18	8	74	
2004	A	991,400 (58,200)	26	9	65	
2008	A	1,644,500 (117,900)	17	10	72	
2012	A	815,900 (69,500)	15	9	77	
2017	A	746,500 (89,800)	2	2	96	

[1] Assumed a coefficient of variation of 50% (see Hammill et al 2021).

Table S2. Total annual number of harp seal females sampled (N) for pregnancy determination and number of females that were pregnant at the time of sampling (Preg). Detection of pregnancy in sampled females was performed as per Stenson et al. (2016). Briefly, females were classified as immature if the ovaries were small and contained only inactive follicles with an absence of *corpus luteum* (CL) or *corpus albicans* (CA) (Fisher 1964; Bowen et al. 1981). Evidence of CL or CA would suggest that the female was sexually mature at the time of sampling. A sexually mature female was deemed pregnant if the presence of a large and fully luteinized CL in one of the ovaries and a foetus in the uterus was detected. Ages were determined to the nearest year by sectioning a lower canine tooth and counting dentine annuli (Fisher 1954; Frie et al. 2011), and the last category (8+) includes ages 8yrs and older.

Year	Age	N	Preg	Year	Age	N	Preg	Year	Age	N	Preg	Year	Age	N	Preg
1954	4	4	0	1969	4	25	1	1982	4	4	0	1990	4	8	0
	5	3	1		5	25	4		5	5	2		5	6	1
	6	3	2		6	16	7		6	1	1		6	3	1
	7	16	12		7	28	23		7	4	3		7	1	0
	8+	33	29		8+	165	146		8+	3	1		8+	10	6
1964	4	11	0	1970	4	13	0	1985	4	4	0	1991	4	10	0
	5	9	1		5	13	3		5	3	1		5	11	2
	6	2	1		6	12	6		6	5	2		6	7	4
	7	4	3		7	10	9		7	3	3		7	3	1
	8+	25	22		8+	107	92		8+	1	1		8+	29	18
1965	4	30	1	1978	4	40	1	1986	4	1	1	1992	4	9	2
	5	44	5		5	38	23		5	0	NA		5	11	3
	6	37	20		6	20	18		6	2	1		6	7	3
	7	38	27		7	9	6		7	1	0		7	8	6
	8+	109	96		8+	0	NA		8+	11	8		8+	32	21
1966	4	7	0	1979	4	4	1	1987	4	12	2	1993	4	11	0
	5	9	1		5	1	1		5	8	3		5	17	2
	6	17	6		6	0	NA		6	9	7		6	7	0
	7	11	8		7	1	1		7	4	4		7	5	4
	8+	49	43		8+	8	4		8+	24	15		8+	35	16
1967	4	10	0	1980	4	2	0	1988	4	17	2	1994	4	23	1
	5	19	4		5	2	1		5	6	1		5	15	2
	6	33	20		6	1	1		6	3	3		6	14	6
	7	29	28		7	0	NA		7	0	NA		7	6	2
	8+	123	109		8+	12	9		8+	19	14		8+	40	33
1968	4	27	0	1981	4	5	1	1989	4	8	0	1995	4	10	0
	5	19	6		5	4	3		5	9	0		5	13	6
	6	20	14		6	2	1		6	6	2		6	4	2
	7	12	11		7	7	6		7	3	2		7	5	2
	8+	55	48		8+	17	14		8+	25	21		8+	26	14

Table S2 *(continued).*

Year	Age	N	Preg	Year	Age	N	Preg	Year	Age	N	Preg	Year	Age	N	Preg
1996	4	8	0	2002	4	2	0	2008	4	6	0	2014	4	2	0
	5	6	0		5	4	1		5	3	0		5	0	NA
	6	4	1		6	5	3		6	2	0		6	1	0
	7	1	1		7	16	9		7	0	NA		7	1	0
	8+	37	24		8+	71	30		8+	61	43		8+	76	65
1997	4	5	0	2003	4	1	0	2009	4	1	0	2015	4	0	NA
	5	4	0		5	3	2		5	1	0		5	1	0
	6	10	3		6	2	1		6	1	0		6	0	NA
	7	2	2		7	3	2		7	1	1		7	3	0
	8+	36	26		8+	90	57		8+	105	59		8+	19	15
1998	4	6	0	2004	4	2	0	2010	4	0	NA	2016	4	7	0
	5	10	3		5	5	0		5	0	NA		5	4	1
	6	9	2		6	5	1		6	0	NA		6	6	2
	7	4	2		7	1	0		7	1	0		7	4	3
	8+	36	21		8+	77	23		8+	114	35		8+	93	69
1999	4	6	0	2005	4	9	1	2011	4	3	0	2017	4	7	0
	5	7	0		5	9	0		5	2	0		5	8	0
	6	17	4		6	13	2		6	0	NA		6	0	NA
	7	15	6		7	7	0		7	0	NA		7	2	0
	8+	60	35		8+	86	54		8+	153	30		8+	50	29
2000	4	1	0	2006	4	2	0	2012	4	2	0	2018	4	10	0
	5	9	3		5	0	NA		5	1	0		5	6	0
	6	6	4		6	0	NA		6	0	NA		6	3	1
	7	5	2		7	0	NA		7	0	NA		7	2	1
	8+	42	29		8+	119	57		8+	12	5		8+	69	51
2001	4	2	0	2007	4	1	0	2013	4	1	0	2019	4	5	0
	5	0	NA		5	5	0		5	0	NA		5	4	0
	6	2	2		6	3	1		6	0	NA		6	2	0
	7	3	0		7	2	2		7	1	0		7	4	1
	8+	39	26		8+	84	62		8+	11	6		8+	110	80

Table S3. Number of harp seals observed in each age class, starting at age 5. Age class 36 represents a multi-year class comprised of all animals older than 35 years of age. Reproductive tracts and jaws were collected between October-February, 1979-2019, around Newfoundland and southern Labrador by Department of Fisheries and Oceans (DFO) personnel and experienced seal hunters under licenses issued by DFO.

Age	1979	1980	1981	1982	1983	1984	1985	1986	1987	1988	1989	1990	1991	1992	1993	1994	1995	1996	1997	1998	1999
5	41	57	48	69	16	15	12	28	38	28	32	24	43	21	42	42	24	34	8	32	23
6	16	41	41	31	6	8	9	28	41	19	20	18	21	19	29	34	11	23	11	36	28
7	18	36	30	28	5	12	6	15	34	11	6	11	7	13	18	19	12	12	5	19	21
8	12	28	26	19	2	6	7	12	14	13	4	3	6	8	18	18	4	2	3	20	13
9	9	15	12	12	4	4	3	12	18	8	8	5	7	11	13	14	3	1	2	10	22
10	7	11	18	5	4	6	4	6	11	7	2	5	4	5	6	10	2	8	7	1	5
11	5	10	14	13	2	4	0	7	14	4	2	8	10	5	7	9	3	8	7	4	7
12	4	8	12	3	1	6	0	7	7	5	2	3	10	4	4	6	0	3	3	3	11
13	6	11	8	5	4	4	1	4	8	8	6	3	5	3	9	6	1	4	3	4	9
14	6	10	9	7	2	5	4	6	7	4	3	3	4	3	4	4	2	6	1	6	8
15	3	10	18	10	2	4	3	5	9	7	5	1	7	0	3	10	2	3	2	5	5
16	4	9	11	4	0	2	0	5	6	6	4	3	3	4	4	2	2	9	4	1	5
17	7	10	12	11	4	1	0	2	8	3	3	1	3	5	4	8	4	10	1	3	2
18	6	0	9	2	1	2	1	6	5	5	4	5	2	4	1	8	6	3	2	2	5
19	5	2	9	10	2	2	1	4	3	6	2	1	2	10	2	3	4	3	0	0	5
20	3	11	8	6	2	2	2	5	11	5	3	5	3	6	2	2	6	3	7	2	5
21	2	7	9	9	1	3	0	8	7	0	4	3	2	3	8	5	3	4	3	3	0
22	3	8	6	0	1	1	0	4	2	0	5	3	4	2	4	4	1	3	3	0	0
23	1	4	3	6	2	3	0	6	7	1	4	5	0	3	2	3	4	2	2	2	3
24	3	1	4	3	1	2	0	4	6	5	2	0	3	6	4	4	0	5	2	2	2
25	3	3	0	2	0	1	0	4	6	6	0	4	2	5	1	0	0	2	0	1	1
26	1	4	1	2	0	1	0	0	4	3	0	1	2	3	4	0	1	3	0	2	0
27	1	1	1	2	0	0	2	2	7	1	1	2	1	1	2	0	1	1	1	1	1
28	0	3	3	3	0	1	1	2	4	0	1	2	5	1	4	3	1	2	0	2	2
29	0	1	1	3	1	1	0	2	3	1	1	0	1	2	1	1	0	0	0	0	2
30	0	3	2	2	0	0	1	0	1	3	0	1	3	1	4	1	0	0	2	0	1
31	0	0	0	0	0	0	0	2	8	1	0	2	3	3	1	0	1	0	1	1	2
32	1	0	0	0	0	0	0	1	2	0	0	0	2	1	0	1	1	1	0	1	0
33	0	1	0	0	0	0	0	0	3	0	0	0	0	0	1	0	0	0	0	0	0
34	0	0	0	0	0	0	0	3	0	1	0	0	2	0	0	0	0	0	0	0	1
35	0	0	0	0	0	0	0	0	1	0	0	0	1	3	0	0	0	1	0	0	0
36	0	1	0	1	0	0	0	0	2	0	0	0	1	5	0	1	0	0	0	0	1

Table S3 *(continued).*

Age	2000	2001	2002	2003	2004	2005	2006	2007	2008	2009	2010	2011	2012	2013	2014	2015	2016	2017	2018	2019
5	15	4	6	11	7	11	4	5	5	1	1	5	2	1	1	1	20	8	1	1
6	14	6	8	10	9	16	5	3	3	1	2	2	0	0	2	1	18	2	2	0
7	7	9	20	6	3	8	0	2	0	1	1	1	0	1	2	4	8	0	0	2
8	11	9	8	5	13	7	8	2	4	1	0	0	0	0	2	0	9	1	0	0
9	7	7	18	8	8	5	8	5	3	1	1	0	0	4	4	0	4	0	0	0
10	10	10	5	11	2	11	15	4	3	3	0	0	0	0	0	0	2	0	0	0
11	6	7	6	4	12	4	7	4	6	1	2	1	0	0	2	0	1	0	0	0
12	6	6	6	13	7	8	13	5	1	5	1	1	0	0	2	2	1	0	0	1
13	5	4	10	14	8	8	18	8	1	7	2	3	0	0	0	1	3	0	0	1
14	5	4	7	18	7	17	17	13	3	4	0	5	1	0	1	0	1	0	0	0
15	4	8	6	9	12	3	23	7	11	11	9	7	0	0	1	0	1	0	0	0
16	3	6	6	7	11	13	22	17	7	10	11	4	1	0	1	2	2	0	0	0
17	2	2	0	10	15	14	18	10	6	16	10	14	2	1	0	0	3	0	0	0
18	4	1	2	5	9	12	17	7	6	18	17	11	3	2	2	0	5	0	1	3
19	2	2	2	9	8	5	13	15	3	7	17	16	2	0	4	0	4	2	0	0
20	2	2	6	2	11	6	15	7	8	16	15	25	6	1	8	1	9	4	0	1
21	1	1	2	3	3	3	12	5	3	13	19	25	2	1	7	1	7	1	0	2
22	1	2	2	1	5	4	0	4	3	6	22	30	6	1	14	0	13	4	0	4
23	1	2	0	2	6	1	5	3	0	5	10	22	6	2	9	3	12	1	0	4
24	3	2	2	0	3	3	4	2	2	10	15	22	4	2	14	1	16	4	0	4
25	0	1	2	1	3	3	2	2	4	11	16	25	7	0	14	11	29	6	2	7
26	1	0	1	1	1	0	3	1	0	7	9	16	6	0	11	2	27	3	0	5
27	0	1	0	1	2	2	2	1	1	1	3	15	2	1	16	2	16	1	0	4
28	0	1	2	0	1	0	3	0	1	5	6	13	4	1	8	1	13	3	0	7
29	0	0	0	0	1	0	3	1	0	2	0	5	2	1	9	0	10	0	1	0
30	0	0	1	0	3	0	1	1	1	2	4	9	2	0	7	2	10	4	1	6
31	1	1	0	3	1	0	0	0	0	0	2	5	0	0	5	2	2	0	1	6
32	0	0	0	1	0	0	0	0	0	1	0	3	1	0	0	0	2	1	1	4
33	1	0	0	0	0	0	0	0	0	1	4	1	0	0	3	0	2	0	0	3
34	0	0	0	0	0	0	0	0	1	0	2	0	0	0	0	0	1	1	0	0
35	0	0	0	0	1	0	0	0	0	0	0	0	0	0	0	0	4	1	0	0
36	0	0	0	0	2	1	1	0	0	0	0	0	0	0	0	0	0	0	0	0

Table S4. Reported total removals and YOY removals for the 4 sources of human-related mortality. We note that YOY catch data were in some cases reported as numbers of animals and in others reported as proportions of total; in the latter case, proportions were converted to integer numbers (total reported removals × proportion YOY) for presentation and model fitting.

	Reported total removals				Reported YOY removals			
Year	Arctic	Bycatch	Canadian	Greenland	Arctic	Bycatch	Canadian	Greenland
1952	1784	0	307108	16400	89	0	198063	9676
1953	1784	0	272886	16400	89	0	197975	9676
1954	1784	0	264416	19150	89	0	175034	11299
1955	1784	0	333369	15534	89	0	252297	9165
1956	1784	0	389410	10973	89	0	341397	6474
1957	1784	0	245480	12884	89	0	165438	7602
1958	1784	0	297786	16885	89	0	140996	9962
1959	1784	0	320134	8928	89	0	238832	5268
1960	1784	0	277350	16154	89	0	156168	9531
1961	1784	0	187866	11996	89	0	168819	7078
1962	1784	0	319989	8500	89	0	207088	5015
1963	1784	0	342042	10111	89	0	270419	5864
1964	1784	0	341663	9203	89	0	266382	5338
1965	1784	0	234253	9289	89	0	182758	5388
1966	1784	0	323139	7057	89	0	251135	4093
1967	1784	0	334356	4242	89	0	277750	2460
1968	1784	0	192696	7116	89	0	156458	4127
1969	1784	0	288812	6438	89	0	233340	3734
1970	1784	77	257495	6269	89	60	217431	3310
1971	1784	525	230966	5572	89	440	210579	3502
1972	1784	622	129883	5994	89	481	116810	3431
1973	1784	468	123832	9212	89	361	98335	5091
1974	1784	183	147635	7145	89	141	114825	4597
1975	1784	286	174363	6752	89	220	140638	4165
1976	1784	1095	165002	11956	89	926	132085	7209
1977	1784	1633	155143	12866	89	1324	126982	9899
1978	2129	3376	161723	16638	106	2763	116190	6981
1979	3620	3603	160541	17545	181	3031	132458	8842
1980	6350	2814	169526	15255	318	2540	132421	4022
1981	4672	4181	202169	22974	234	3775	178394	6057
1982	4881	3817	166739	26927	244	3470	145274	8280
1983	4881	5009	57889	24785	244	4547	50058	6760
1984	4881	4143	31544	25829	244	3714	23922	3686
1985	4881	4987	19035	20785	244	4345	13334	5197
1986	4881	6109	25934	26099	244	5213	21888	6526
1987	4881	10911	46796	37859	244	9047	36350	9467
1988	4881	8399	94046	40415	244	6993	66972	10106
1989	4881	8644	65304	42971	244	7918	56346	10745

Table S4. *(continued)*.

	Reported total removals				Reported YOY removals			
Year	Arctic	Bycatch	Canadian	Greenland	Arctic	Bycatch	Canadian	Greenland
1990	4881	2769	60162	45526	244	1974	34402	11384
1991	4881	8702	52588	48082	244	8094	42382	12023
1992	4881	23035	68668	50638	244	16624	43866	12662
1993	4881	26976	27003	56319	244	19244	16401	14083
1994	4881	47604	61379	57373	244	36768	25223	14346
1995	4881	20593	65767	62749	244	14252	34106	15691
1996	4881	29641	242906	73947	244	10896	184856	18491
1997	2500	19048	264210	68816	125	13860	220476	17208
1998	1000	4557	282624	81262	50	3584	251403	20320
1999	500	16168	244552	93117	25	9843	237644	23284
2000	400	11522	92055	98463	20	9890	85035	24621
2001	600	20064	226493	85428	30	15072	214754	21361
2002	1000	9543	312367	66735	50	5642	297764	16687
2003	1000	5445	289512	66149	50	3533	280174	16541
2004	1000	35870	365971	70587	50	24642	353553	17650
2005	1000	26378	329829	91688	50	18094	323800	22927
2006	1000	21656	354867	94034	50	16130	346426	23513
2007	1000	9450	224745	82826	50	6460	221488	20711
2008	1000	7280	217850	80444	50	4920	217565	20115
2009	1000	2275	76688	71862	50	1303	76688	17969
2010	1000	3956	69101	89905	50	2618	68654	22481
2011	1000	2114	40389	73462	50	1402	40371	18369
2012	1000	2886	71460	54660	50	2074	71319	13668
2013	1000	177	97922	65241	50	150	94310	16314
2014	1000	1166	59666	63028	50	952	59616	15760
2015	1000	1040	35382	61767	50	844	35302	15445
2016	1000	603	68360	55520	50	464	61016	13883
2017	1000	226	81742	47516	50	173	70270	11881
2018	1000	611	61022	47014	50	450	56135	11756
2019	1000	711	32038	48824	50	563	29913	12209

Table S5. Levels of uncertainty in the total number of catches and the proportion of young-of-the-year (YOY) in different sources of human-related mortality (Arctic hunt, Bycatch, Canadian hunt, Greenland hunt). For total catches, uncertainty was quantified as the coefficient of variation (CV) associated with each reported number. For proportion YOY, uncertainty was quantified as parameter κ, the precision parameter of the beta distribution (see Supplementary Methods above, and main text equation 31). Values of κ were set to 25, 100 or 2500 (for low, medium or high certainty, respectively), which correspond to standard deviations for a proportion of 0.5 of 0.1, 0.05 and 0.01.

	Total catches, coefficient of variation (CV)				Proportion YOY, κ (higher = more precise)			
Year	Arctic	Bycatch	Canadian	Greenland	Arctic	Bycatch	Canadian	Greenland
1952	0.2	0.1	0.1	0.1	25	2500	100	100
1953	0.2	0.1	0.1	0.1	25	2500	100	100
1954	0.2	0.1	0.1	0.05	25	2500	100	100
1955	0.2	0.1	0.1	0.05	25	2500	100	100
1956	0.2	0.1	0.1	0.05	25	2500	100	100
1957	0.2	0.1	0.1	0.05	25	2500	100	100
1958	0.2	0.1	0.1	0.05	25	2500	100	100
1959	0.2	0.1	0.1	0.05	25	2500	100	100
1960	0.2	0.1	0.1	0.05	25	2500	100	100
1961	0.2	0.1	0.1	0.05	25	2500	100	100
1962	0.2	0.1	0.1	0.05	25	2500	100	100
1963	0.2	0.1	0.1	0.05	25	2500	100	100
1964	0.2	0.1	0.1	0.05	25	2500	100	100
1965	0.2	0.1	0.1	0.05	25	2500	2500	100
1966	0.2	0.1	0.1	0.05	25	2500	2500	100
1967	0.2	0.1	0.1	0.05	25	2500	2500	100
1968	0.2	0.1	0.1	0.05	25	2500	2500	100
1969	0.2	0.1	0.1	0.05	25	2500	2500	100
1970	0.2	0.1	0.1	0.05	25	2500	2500	2500
1971	0.2	0.1	0.05	0.05	25	2500	2500	2500
1972	0.2	0.1	0.05	0.05	25	2500	2500	2500
1973	0.2	0.1	0.05	0.05	25	2500	2500	2500
1974	0.2	0.1	0.05	0.05	25	2500	2500	2500
1975	0.2	0.1	0.05	0.05	25	2500	2500	2500
1976	0.2	0.1	0.05	0.05	25	2500	2500	2500
1977	0.2	0.1	0.05	0.05	25	2500	2500	2500
1978	0.2	0.1	0.05	0.05	25	2500	2500	2500
1979	0.2	0.1	0.05	0.05	25	2500	2500	2500
1980	0.2	0.1	0.05	0.05	25	2500	2500	2500
1981	0.2	0.1	0.05	0.05	25	2500	2500	2500
1982	0.2	0.1	0.05	0.05	25	2500	2500	2500
1983	0.2	0.1	0.05	0.05	25	2500	2500	2500
1984	0.2	0.1	0.05	0.05	25	2500	2500	25
1985	0.2	0.1	0.05	0.05	25	2500	2500	25

Table S5 *(continued).*

	Total catches, coefficient of variation (CV)				Proportion YOY, κ (higher = more precise)			
Year	Arctic	Bycatch	Canadian	Greenland	Arctic	Bycatch	Canadian	Greenland
1986	0.2	0.1	0.05	0.05	25	2500	2500	25
1987	0.2	0.1	0.05	0.05	25	2500	2500	25
1988	0.2	0.1	0.05	0.2	25	2500	2500	25
1989	0.2	0.05	0.05	0.2	25	2500	2500	25
1990	0.2	0.05	0.05	0.2	25	2500	2500	25
1991	0.2	0.05	0.05	0.2	25	2500	2500	25
1992	0.2	0.05	0.05	0.2	25	2500	2500	25
1993	0.2	0.05	0.05	0.1	25	2500	2500	25
1994	0.2	0.05	0.05	0.1	25	2500	2500	25
1995	0.2	0.05	0.05	0.1	25	2500	2500	25
1996	0.2	0.05	0.05	0.1	25	2500	2500	25
1997	0.2	0.05	0.05	0.1	25	2500	2500	25
1998	0.2	0.05	0.05	0.1	25	2500	2500	25
1999	0.2	0.05	0.05	0.1	25	2500	2500	25
2000	0.2	0.05	0.05	0.1	25	2500	2500	25
2001	0.2	0.05	0.05	0.1	25	2500	2500	25
2002	0.2	0.05	0.05	0.1	25	2500	2500	25
2003	0.2	0.05	0.05	0.1	25	2500	2500	25
2004	0.2	0.1	0.05	0.1	25	2500	2500	25
2005	0.2	0.1	0.05	0.1	25	2500	2500	25
2006	0.2	0.1	0.05	0.1	25	2500	2500	25
2007	0.2	0.1	0.05	0.1	25	2500	2500	25
2008	0.2	0.1	0.05	0.1	25	2500	2500	25
2009	0.2	0.1	0.05	0.1	25	2500	2500	25
2010	0.2	0.1	0.05	0.1	25	2500	2500	25
2011	0.2	0.1	0.05	0.1	25	2500	2500	25
2012	0.2	0.1	0.05	0.1	25	2500	2500	25
2013	0.2	0.1	0.05	0.1	25	2500	2500	25
2014	0.2	0.1	0.05	0.1	25	2500	2500	25
2015	0.2	0.1	0.05	0.1	25	2500	2500	25
2016	0.2	0.1	0.05	0.1	25	2500	2500	25
2017	0.2	0.1	0.05	0.1	25	2500	2500	25
2018	0.2	0.1	0.05	0.1	25	2500	2500	25
2019	0.2	0.1	0.05	0.1	25	2500	2500	25

Table S6. Summary of process model parameters, including brief descriptions, prior probability distributions, and parameters for prior distributions. Prior distributions in bold font indicate those that were moderately informed by previous information (see SI Methods for details); all other priors are vague and effectively uninformative with respect to the system, although scaled according to basic biological expectations. Note that the units for initial population size (N_0) are millions of animals. For human removal parameters having j subscripts ($\sigma_{H,j}$, $\bar{\gamma}_{H0,j}$, $\bar{\gamma}_{HA,j}$), subscript 1 = arctic hunt, subscript 2 = bycatch, subscript 3 = Canadian commercial harvest, and subcript 4 = Greenland harvest.

Parameter	Description	Prior distrib.	param1	Param2
N_0	Initial abundance (excluding YOY)	Half-Cauchy	0	0.5
α_0	Age-based hazards: 1-yr old juvenile	Half-Cauchy	0	0.1
α_1	Age effect on natural hazards, early life	Half-Cauchy	0	0.1
α_2	Age effect on natural hazards, late life	Half-Cauchy	0	0.1
υ	Additional hazards for YOY (vs. 1 yr old)	**Normal**	**log(3)**	**0.2**
β_1	Baseline fecundity, 8+ yr old females	Half-Cauchy	0	0.1
β_2	Age effect on fecundity, 4-8 yr olds	Half-Cauchy	0	0.1
ϕ_F	Density-dependent effects, fecundity	Half-Cauchy	0	0.1
ϕ_S	Density-dependent effects, survival	Half-Cauchy	0	0.1
ζ	Scaling ratio for density-dependent and environmental effects, adult vs YOY	**Beta**	**0.1*50**	**0.9*50**
δ	Effects of environmental variation (NLCI)	Cauchy	0	0.1
ψ_0	Effects of ice anomalies, intercept logit fxn.	**Normal**	**3**	**1.5**
ψ_{Gulf}	Effects of ice anomalies, slope of logit fxn.	**Normal**	**1.5**	**1**
ψ_{Front}	Effects of ice anomalies, slope of logit fxn.	**Normal**	**1.5**	**1**
σ_S	Magnitude env. Stochasticity, survival	Half-Cauchy	0	0.1
σ_F	Magnitude env. Stochasticity, fecundity	Half-Cauchy	0	0.1
τ	Precision param for age distributions	Half-Cauchy	0	5
η	Precision param for pregnancy proportions	Half-Cauchy	0	2.5
$\sigma_{H,1}$	Magnitude variance in human removals j=1	Half-Cauchy	0	0.1
$\sigma_{H,2}$	Magnitude variance in human removals j=2	Half-Cauchy	0	0.1
$\sigma_{H,3}$	Magnitude variance in human removals j=3	Half-Cauchy	0	0.1
$\sigma_{H,4}$	Magnitude variance in human removals j=4	Half-Cauchy	0	0.1
$\bar{\gamma}_{H0,1}$	Mean log hazard, YOY removals, j=1	Half-Cauchy	0	1
$\bar{\gamma}_{H0,2}$	Mean log hazard, YOY removals, j=2	Half-Cauchy	0	1
$\bar{\gamma}_{H0,3}$	Mean log hazard, YOY removals, j=3	Half-Cauchy	0	1
$\bar{\gamma}_{H0,4}$	Mean log hazard, YOY removals, j=4	Half-Cauchy	0	1
$\bar{\gamma}_{HA,1}$	Mean log hazard, adult removals, j=1	Half-Cauchy	0	1
$\bar{\gamma}_{HA,2}$	Mean log hazard, adult removals, j=2	Half-Cauchy	0	1
$\bar{\gamma}_{HA,3}$	Mean log hazard, adult removals, j=3	Half-Cauchy	0	1
$\bar{\gamma}_{HA,4}$	Mean log hazard, adult removals, j=4	Half-Cauchy	0	1

Table S7. Summary of parameter estimates from model fitting, including the mean, standard deviation and lower/upper 95% quantiles of the posterior distributions. Also shown are the effective sample size and r-hat statistics for each parameter, with values close to 1.0 indicating well mixed chains (and thus model cnvergence). Note that the units for initial population size (N_0) are millions of animals. For human removal parameters having j subscripts ($\sigma_{H,j}$, $\bar{\gamma}_{H0,j}$, $\bar{\gamma}_{HA,j}$), subscript 1 = arctic hunt, subscript 2 = bycatch, subscript 3 = Canadian commercial harvest, and subcript 4 = Greenland harvest.

Parameter	Mean	SD	CI95_lo	CI95_hi	N effect	r-hat
N_0	2.085	0.128	1.885	2.297	3101	1.001
α_0	5.844	0.396	5.128	6.412	1807	1.005
α_1	0.263	0.055	0.177	0.358	3607	1.001
α_2	0.010	0.002	0.008	0.013	3395	1.001
υ	1.074	0.200	0.749	1.410	4605	0.999
β_1	1.239	0.148	0.992	1.473	3026	1.002
β_2	0.146	0.008	0.132	0.160	3562	1.002
ϕ_F	0.232	0.043	0.165	0.308	2651	1.003
ϕ_S	0.627	0.094	0.479	0.786	2173	1.003
ζ	0.047	0.028	0.014	0.099	3202	1.001
δ	0.413	0.077	0.282	0.533	3287	1.002
ψ_0	2.830	1.040	1.159	4.604	4285	1.001
ψ_{Gulf}	0.902	0.694	-0.331	1.990	4457	1.001
ψ_{Front}	2.322	0.679	1.170	3.352	3867	1.003
σ_S	0.183	0.078	0.078	0.326	1334	1.007
σ_F	0.325	0.081	0.210	0.467	2181	1.002
τ	144.8	13.9	122.9	168.7	4325	1.000
η	14.3	3.9	9.2	21.4	4253	1.000
$\sigma_{H,1}$	0.807	0.069	0.700	0.925	2088	1.004
$\sigma_{H,2}$	4.425	0.372	3.879	5.082	925	1.007
$\sigma_{H,3}$	1.801	0.118	1.618	2.001	1275	1.003
$\sigma_{H,4}$	0.562	0.049	0.485	0.645	1704	1.004
$\bar{\gamma}_{H0,1}$	2.456	0.144	2.218	2.691	3900	0.999
$\bar{\gamma}_{H0,2}$	2.016	0.579	1.038	2.947	231	1.039
$\bar{\gamma}_{H0,3}$	8.967	0.245	8.575	9.380	349	1.018
$\bar{\gamma}_{H0,4}$	7.031	0.086	6.890	7.172	1691	1.003
$\bar{\gamma}_{HA,1}$	3.373	0.108	3.190	3.545	1779	1.004
$\bar{\gamma}_{HA,2}$	0.232	0.193	0.015	0.624	579	1.015
$\bar{\gamma}_{HA,3}$	5.371	0.221	5.006	5.726	363	1.030
$\bar{\gamma}_{HA,4}$	5.521	0.080	5.390	5.660	1640	1.004
